# Thrombin induces ACSL4-dependent ferroptosis during cerebral ischemia/reperfusion

**DOI:** 10.1038/s41392-022-00917-z

**Published:** 2022-02-23

**Authors:** Qing-zhang Tuo, Yu Liu, Zheng Xiang, Hong-Fa Yan, Ting Zou, Yang Shu, Xu-long Ding, Jin-jun Zou, Shuo Xu, Fei Tang, Yan-qiu Gong, Xiao-lan Li, Yu-jie Guo, Zhao-yue Zheng, Ai-ping Deng, Zhang-zhong Yang, Wen-jing Li, Shu-ting Zhang, Scott Ayton, Ashley I. Bush, Heng Xu, Lunzhi Dai, Biao Dong, Peng Lei

**Affiliations:** 1 Department of Geriatrics and State Key Laboratory of Biotherapy, National Clinical Research Center for Geriatrics, West China Hospital, Sichuan University, 610041 Chengdu, Sichuan China; 2 Department of Neurology and State Key Laboratory of Biotherapy, West China Hospital, Sichuan University, 610041 Chengdu, Sichuan China; 3 West China School of Basic Medical Sciences and Forensic Medicine, Sichuan University, 610041 Chengdu, Sichuan China; 4 Department of Laboratory Medicine, Precision Medicine Center, State Key Laboratory of Biotherapy, West China Hospital, Sichuan University, 610041 Chengdu, Sichuan China; 5 Melbourne Dementia Research Centre, Florey Institute of Neuroscience and Mental Health, The University of Melbourne, Parkville, VIC 3010 Australia

**Keywords:** Diseases of the nervous system, Molecular neuroscience

## Abstract

Ischemic stroke represents a significant danger to human beings, especially the elderly. Interventions are only available to remove the clot, and the mechanism of neuronal death during ischemic stroke is still in debate. Ferroptosis is increasingly appreciated as a mechanism of cell death after ischemia in various organs. Here we report that the serine protease, thrombin, instigates ferroptotic signaling by promoting arachidonic acid mobilization and subsequent esterification by the ferroptotic gene, acyl-CoA synthetase long-chain family member 4 (ACSL4). An unbiased multi-omics approach identified thrombin and ACSL4 genes/proteins, and their pro-ferroptotic phosphatidylethanolamine lipid products, as prominently altered upon the middle cerebral artery occlusion in rodents. Genetically or pharmacologically inhibiting multiple points in this pathway attenuated outcomes of models of ischemia in vitro and in vivo. Therefore, the thrombin-ACSL4 axis may be a key therapeutic target to ameliorate ferroptotic neuronal injury during ischemic stroke.

## Introduction

Stroke remains one of humanity’s biggest killers, including in developing countries.^1–3^ Ischemic stroke, which is the most common form,^4^ occurs as a result of a blood clot occluding blood vessels that serve the brain, which causes the brain to lose energy and oxygen supply. The resulting brain damage develops from 20 min and can persist up to 10 days post stroke;^5^ however, the consequences of stroke can last for years, where one-third of stroke patients are left permanently disabled.^6^ Restoration of blood flow to the brain, either pharmacologically or by mechanical thrombolysis, is the main management strategy for stroke. Recombinant tissue plasminogen activator (r-tPA) is the only Food and Drug Administration-approved medication, which functions as a serine protease to dissolve clots,^7,8^ and has been shown to promote brain plasticity through epidermal growth factor receptor signaling that leads to longer-term functional improvement.^9^ However, in routine clinical practice, only 11% of patients are eligible to receive r-tPA due to the limited time window after stroke, a moderate recanalization rate, and other clinical contraindications. Moreover, of those who receive r-tPA, clinical improvement is only observed in one-half of patients.^10^ Therefore, other treatments, especially those that can protect against neuronal death, are still urgently needed.

Unfortunately, over the past 10 years, only antioxidants, statins, and anticoagulants have met their primary endpoints in phase III/IV clinical trials despite numerous preclinical discoveries of neuroprotective agents.^11^ This translational “roadblock” highlights the need to further understand the mechanisms that lead to cell damage and death after cerebral infarction. The pathophysiology of ischemic stroke provides possibilities for several cell death mechanisms to occur, which may be promising targets for intervention.^11^ For example, the lack of oxygen and glucose supply during ischemia severely limits ATP production, which is especially necessary for neurons to power the sodium–potassium pump that maintains membrane polarization.^11^ Failure to regulate the membrane potential of the neuron causes manifold toxic cascades including excitotoxicity, mitochondrial dysfunction, local acidosis, protein misfolding, and inflammation. Regarding the excitotoxicity cascade, depolarization of the membrane as a consequence of glucose and oxygen deprivation causes glutamate release that signals for intracellular calcium elevation in postsynaptic neurons that result in cell death by apoptosis and necroptosis.^12^ The ischemic core is the area of the brain most affected by the lesion of the stroke, where there is substantial cell death, whereas the penumbra is the volume surrounding the core that is damaged but has greater potential to be rescued with therapeutics.

If the neurons survive this period of oxygen and glucose starvation, they may be challenged with secondary oxygen stress upon reperfusion. This is why targeting *oxidative* stress is a rational therapeutic approach in a disease that is (initially) complicated by oxygen deprivation. In particular, iron-dependent ferroptosis causes cell death by excess lipid peroxidation that may contribute to the death of neurons in stroke. We previously found that soluble tau protein, previously reported to mediate iron transport,^13–15^ decreases after transient focal ischemia, which contributed to iron elevation and neuronal damage in a mouse model of ischemic stroke.^16^ Inhibition of ferroptosis by ferrostatin-1 and liproxstatin-1 protected the brains from cerebral ischemic injury in mice.^16,17^ In addition, transcriptional changes consistent with ferroptosis have also been identified in hemorrhagic stroke models,^18^ and selenium supplementation to enhance glutathione peroxidase 4 activity, the key checkpoint for ferroptosis, ameliorates ferroptotic damage in ischemic and hemorrhagic stroke models.^18,19^

To investigate the key proteins or pathways that are responsible for ferroptosis initiation during cerebral ischemia/reperfusion (I/R), we applied an integrative multi-omics approach, to survey the key metabolomics altered, and identify the essential primers of ferroptotic damage in ischemic stroke models. We used genetic or pharmacological modifications in rodents to verify the targets, and tested human samples to trace to the source of its alteration. These data may indicate a tractable target to prevent neuronal death after ischemic stroke.

## Results

### Thrombin is upregulated after acute cerebral I/R

**Fig. 1:**
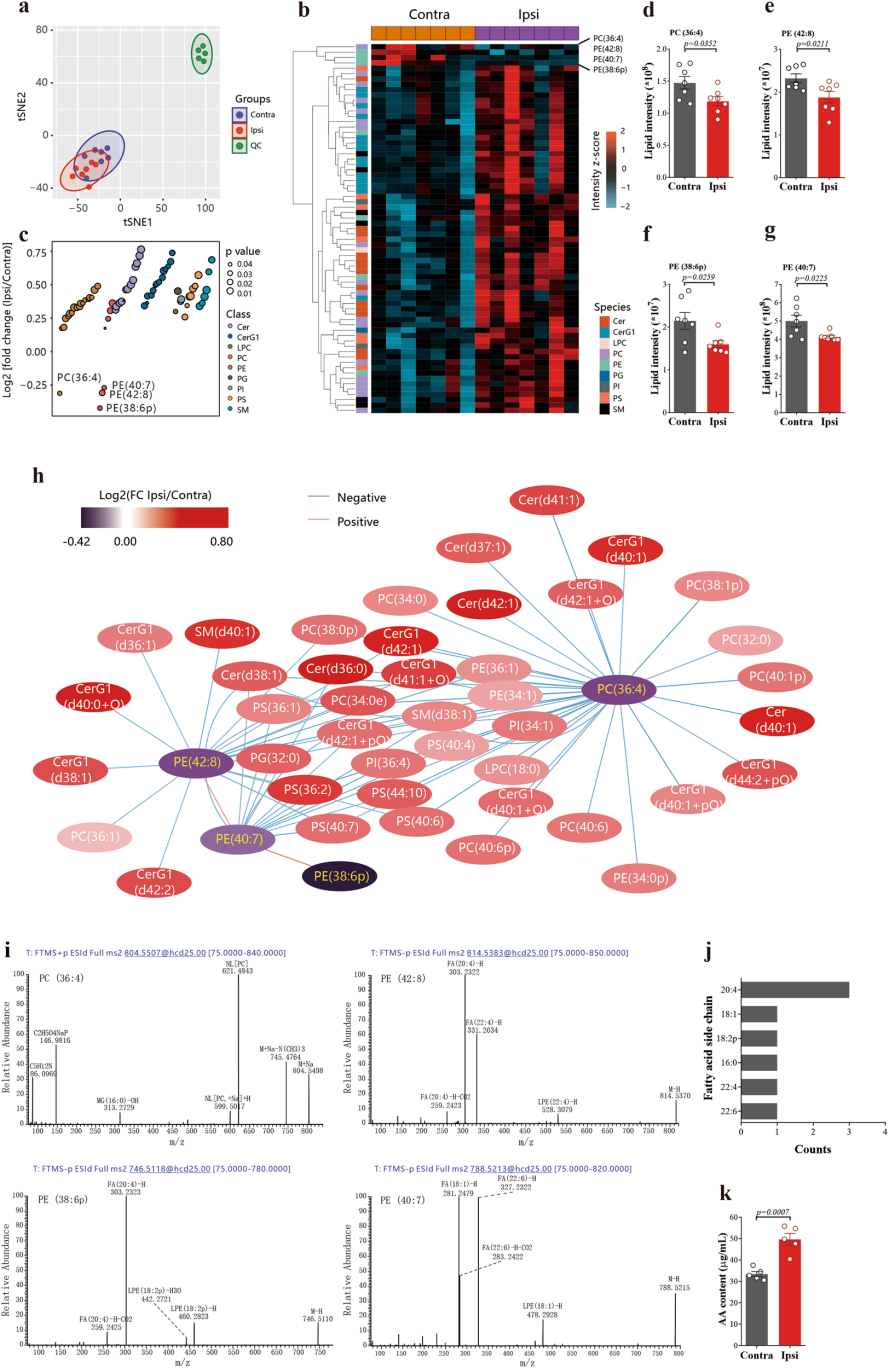
Arachidonic acid on the side chain of phospholipid is released after ischemia/reperfusion. **a** *t*-Distributed Stochastic Neighbor Embedding (tSNE) analysis shows remarkable stability of the instrument and good reproducibility of samples. **b** Heatmap analysis of the different expression (DE) lipids in contralateral (Contra) group and ipsilateral (Ipsi) group. The DE lipids were selected by VIP > 1, which was calculated by OPLS-DA analysis, and *p* value <0.05 (*t* test). **c** Different expression lipids in the Ipsi group. Values are shown as log2 fold change relative to the Contra group. Each dot denotes one lipid type, and the dot color represents lipid class, and the dot size shows significance (*t* test). **d–g** The abundance of PC (36:4, 16:0/20:4), PE (42:8, 22:4/20:4), PE (38:6, 18:2/20:4), and PE (40:7, 18:1/22:6) are significantly downregulated in Ipsi group. Data are mean ± SEM, *n* = 6. *t* test was performed. **h** The correlation among the 69 significantly changed lipids between Contra and Ipsi. The color of a node represents the ratio (Ipsi/Contra) of the lipids. The wathet edge shows a negative, and the orange edge indicates a positive correlation. **i** The ion characteristic fragments for the four reduced lipids. **j** The statistics of the fatty acid side chain of the four reduced lipids. **k** AA contents of the contralateral and ipsilateral hippocampus of mice were assayed 6 h after MCAO/R. Data are means ± SEM, *n* = 5. *t* test was performed

While several studies^11,16,18^ have implicated ferroptosis in ischemic stroke, the mechanisms driving its activation remain unclear. Cell death caused by ferroptosis results from toxic lipid hydroperoxides that accumulate beyond the capacity of cells to detoxify.^20–22^ Arachidonic acid (AA) containing phosphatidylethanolamines (PEs) are particularly vulnerable to ferroptotic peroxidation.^11,21^ Using a high-quality untargeted metabolomic analysis of hippocampal tissue after ischemic stroke in mice (Fig. 1a), we identified four significantly reduced phospholipids, namely PC (36: 4), PE (42: 8), PE (38: 6p), and PE (40: 7) (Fig. 1b–h). Further analysis of their fatty acid side chains revealed that the fatty acid side chains of PC (36: 4, 16: 0/20: 4), PE (42: 8, 22: 4/20: 4), and PE (38: 6p, 18: 2p/20: 4) contain AA (Fig. 1i), and AA (20:4) was the dominant (Fig. 1j), which was consistent with that dramatically increased free AA in the hippocampal tissues after ischemic stroke in mice (stroke/control ratio = 1.49, *p* = 0.0007, Student’s *t* test, Fig. 1k).

**Fig. 2:**
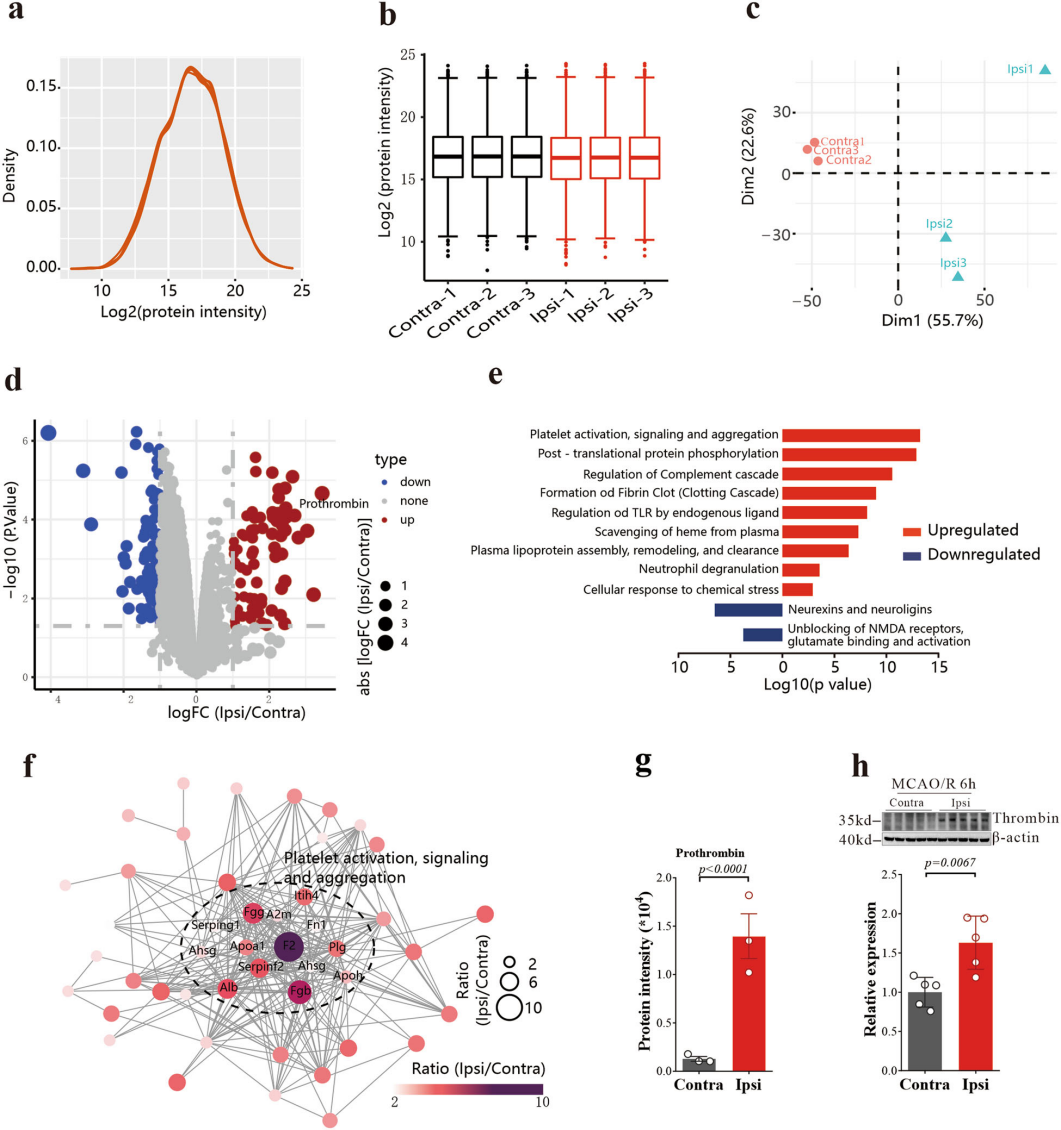
Thrombin is upregulated after acute cerebral ischemia/reperfusion. **a** The unimodal distributions of the protein intensities suggests no obvious degradation in samples. **b** Distribution of log2-transformed intensity of identified proteins in 6 samples. Black presents Contra, and red denotes Ipsi. **c** Principal-component analysis shows a clear separation between Contra (red) and Ipsi (water blue). **d** Sample volcano plot for MCAO mice model showing –log10 (*p* value) and logFC values for all proteins with highlighting for those that are significantly upregulated (red dots) or downregulated (blue dots) after MCAO, and the most changed protein - prothrombin was labeled. Proteins in black are not significantly changed after MCAO. **e** Reactome enrichment for the 75 upregulated (ratio Ipsi/Contra >2, and *p* < 0.05, *t* test) and 111 downregulated (ratio Ipsi/Contra <0.5, and *p* < 0.05, *t* test) proteins, based on the Metascape.^23^ **f** Protein-protein interaction (PPI) analysis for the 75 upregulated proteins. The size and color for the node represent the ratio of Ipsi/Contra. **g** Levels of prothrombin assayed by mass spectrometry in the contralateral and ipsilateral hippocampus of mice 6 h after MCAO/R. Data are means ± SEM, *n* = 3. *t* test was performed. **h** Thrombin protein levels were examined from the contralateral and ipsilateral hippocampus of mice 6 h after MCAO/R. Western blots were analyzed with Image J and normalized to β-actin expression. Data are means ± SEM, *n* = 5. *t* test was performed

To explore the protein responsible for AA elevation after ischemic stroke, we performed proteomic analysis on the same tissue (Fig. 2a–c). Based on the enrichment of the differentially expressed proteins,^23^ we found that platelet activation, signaling, and aggregation was the most upregulated pathway (Fig. 2d, e). Protein-protein interaction (PPI) network analysis also consistently pointed to the same pathway (String, version 11.5) (Fig. 2f), and prothrombin (F2 gene encodes the prothrombin, also known as coagulation factor II), the thrombin proenzyme, was the most upregulated protein (stroke/control ratio = 10.88, *p* < 0.0001, Student’s *t* test, Fig. 2g and Supplementary Fig. S1a), which was further confirmed by western blotting (Fig. 2h).

### Thrombin induces ferroptosis independent of iron accumulation

**Fig. 3:**
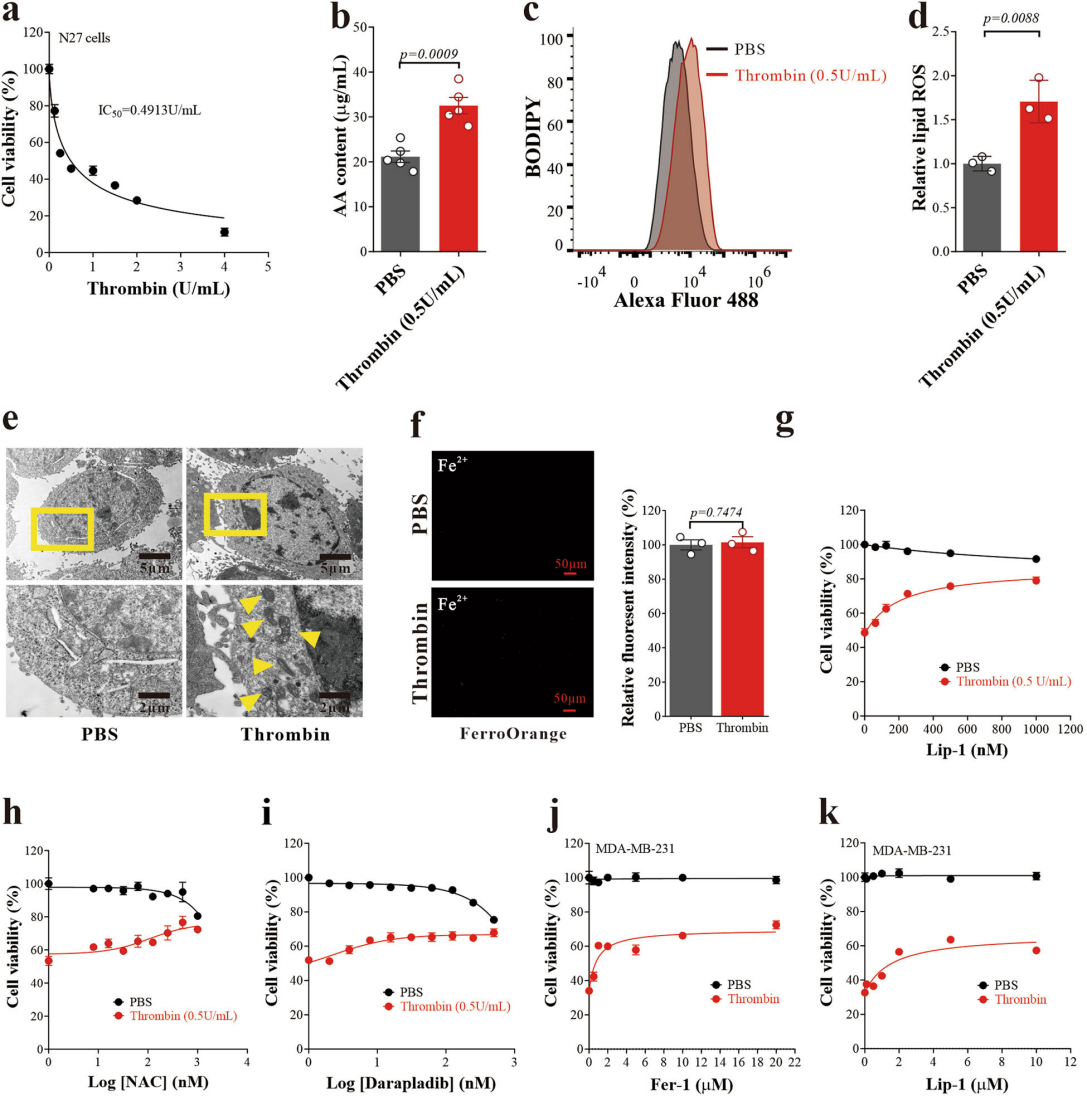
Thrombin induces neuronal ferroptosis. **a** Thrombin cytotoxicity in N27 cells. Data are means ± SEM, *n* = 6 wells from one representative of 3 independent experiments. **b** AA content was assayed from the N27 cells 24 h after thrombin (0.5 U/mL) treatment. Data are means ± SEM, *n* = 5 wells from one representative of 3 independent experiments. *t* test was performed. **c** Lipid ROS in N27 cells treated with thrombin for 24 h (representative histogram plot for fluorescence of oxidized BODIPY-C11). **d** Relative lipid ROS is expressed as the ratio of oxidized to reduced BODIPY-C11 mean fluorescence intensity in N27 cells treated with thrombin for 24 h. Data are means ± SEM, *n* = 3 wells from one representative of 3 independent experiments. *t* test was performed. **e** Transmission electron microscopy (TEM) of N27 cells treated with thrombin (0.5 U/mL) for 24 h. Yellow arrows indicate shrunken mitochondria. **f** Detection of intracellular Fe^2+^ in N27 cells treated with thrombin (0.5 U/mL) for 6 h using FerroOrange. Data are means ± SEM, *n* = 3 wells from one representative of 3 independent experiments. *t* test was performed. **g** Cell viability of N27 cells 24 h after thrombin (0.5 U/mL) and Liproxstatin-1 (Lip-1) of concentration gradient co-treatment. Data are means ± SEM, *n* = 6 wells from one representative of 3 independent experiments. **h**, **i** Cell viability of N27 cells 24 h after thrombin (0.5 U/mL), with NAC (**h**) or Darapladib (**i**) co-treatment. Data are means ± SEM, *n* = 6 wells from one representative of 3 independent experiments. **j**, **k** Cell viability of MDA-MB-231 cells 24 h after thrombin (0.5 U/mL), with Fer-1 (**j**), Lip-1 (**k**) co-treatment. Data are means ± SEM, *n* = 5 wells from one representative of 3 independent experiments

Thrombin is one of the leading drug targets for ischemic stroke, owing to the action of thrombin in stimulating fibrin production and coagulation. However, thrombin has been shown previously to induce apoptosis.^24^ Here, we found that thrombin dose-dependently induced N27 neuronal cell death (Fig. 3a), which accompanied elevated AA (Fig. 3b) and lipid hydroperoxides (Fig. 3c, d), and smaller mitochondria with increased membrane density (Fig. 3e), which are all features consistent with ferroptosis. Thrombin did not affect the intracellular level of ferrous iron, which is not a necessity to induce ferroptosis (Fig. 3f). More importantly, cell death induced by thrombin was prevented by treatment of ferroptosis inhibitors liproxstatin-1 (Fig. 3g) and glutathione precursor *N*-acetyl-L-cysteine (Fig. 3h), and cPLA2α inhibitor darapladib (Fig. 3i). Cell death in MDA-MB-231 cells (a human breast cancer cell line) was likewise prevented by liproxstatin-1, indicating a role for thrombin in ferroptosis outside of the brain (Fig. 3j, k).

One thrombin inhibitor, dabigatran, is currently undergoing phase III clinical trials for ischemic stroke [*ClinicalTrials.gov* Identifier: NCT03961334]. Spontaneously, thrombin, as a serine protease, activates cPLA2α which cleaves AA at the sn-2 position of glycerophospholipids,^25^ which causes AA to be released from the membrane.^11,26–28^ And our data implicate thrombin elevation as an upstream signal for AA mobilization after ischemic stroke, which is known to fuel ferroptosis. Indeed, we found that dabigatran protected cells from the oxygen-glucose deprivation (OGD) model of I/R in N27 neuronal culture (Supplementary Fig. S2b), and relieved neurological symptoms (Supplementary Fig. S2c) and reduced cerebral infarction volume (Supplementary Fig. S2d) in rats after MCAO/Reperfusion (MCAO/R). Since both the OGD and MCAO models do not involve clot formation^11^ that may relate to thrombin inhibition, these findings suggest an additional lesion induced by thrombin that was interdicted by dabigatran. Collectively, these data highlight the possibility for thrombin to induce ferroptosis, which may be involved in ischemic stroke.

### Oxygen-dependent ferroptosis occurred in cerebral I/R injury

**Fig. 4:**
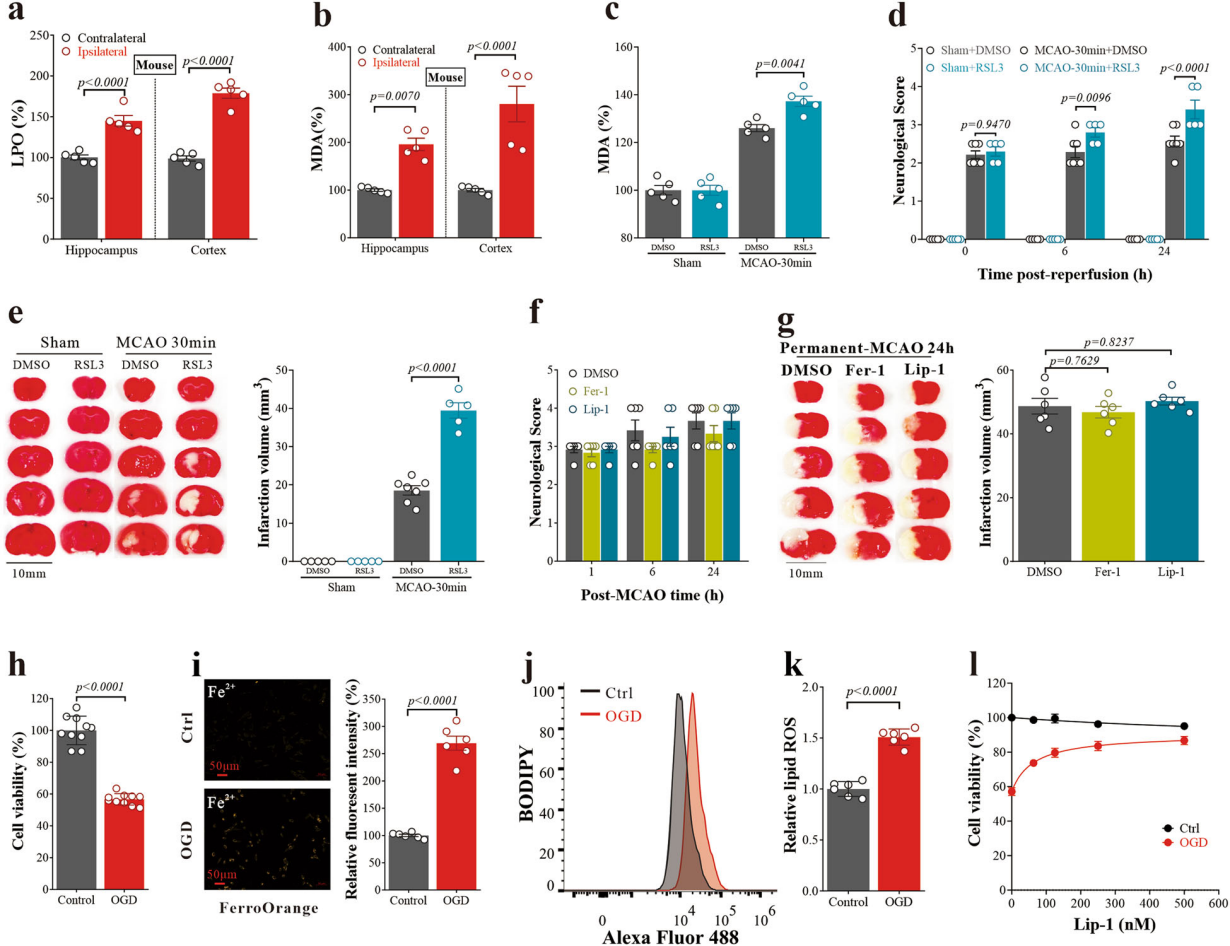
Oxygen-dependent ferroptosis in cerebral reperfusion injury. **a** LPO of the hippocampus and cortex of mice were assayed following MCAO/R for 24 h. Data are means ± SEM, *n* = 5 animals per group. Two-way ANOVA with post hoc Sidak test was performed. **b** MDAs of the hippocampus and cortex of mice were assayed following MCAO/R for 24 h. Data are means ± SEM, *n* = 5 animals per group. Two-way ANOVA with post-hoc Sidak test was performed. **c** MDA was detected in the hippocampus of mice treated with RSL3 following MCAO/R for 24 h. Data are means ± SEM, *n* = 5 animals per group. One-way ANOVA with post-hoc Tukey test was performed. **d** Neurological score (higher numbers indicating more severe impairment) was performed at 0, 6, and 24 h after MCAO/R. Data are means ± SEM. Sham, *n* = 5; MCAO + DMSO, *n* = 7; MCAO + RSL3, *n* = 5. Two-way ANOVA with post-hoc Tukey test was performed. **e** Representative 2,3,5-triphenyl tetrazolium chloride (TTC)-stained serial brain sections of mice 24 h after MCAO/R, where viable tissue stains red. Quantification of infarction volume indicated by TTC staining using Image J. Data are means ± SEM. Sham, *n* = 5; MCAO + DMSO, *n* = 7; MCAO + RSL3, *n* = 5. One-way ANOVA with post-hoc Tukey test was performed. **f** Neurological score was performed at 1, 6, and 24 h after permanent-MCAO. Data are means ± SEM, *n* = 6 animals per group. Two-way ANOVA with post-hoc Tukey test was performed. **g** Representative TTC-stained serial brain sections of mice 24 h after permanent-MCAO, where viable tissue stains red. Quantification of infarction volume indicated by TTC staining using Image J. Data are means ± SEM, *n* = 6 animals per group. One-way ANOVA with post hoc Tukey test was performed. **h** Neuronal cell viability after OGD 2 h/reoxygenation 18 h. Data are means ± SEM, *n* = 10 wells from one representative of 4 independent experiments. *t* test was performed. **i** Detection of intracellular Fe^2+^ in N27 cells using FerroOrange at 2 h of OGD. Data are means ± SEM, *n* = 6 wells from one representative of 3 independent experiments. *t* test was performed. **j** Lipid ROS in N27 cells treated with OGD for 2 h (representative histogram plot for fluorescence of oxidized BODIPY-C11). **k** Relative lipid ROS is expressed as the ratio of oxidized to reduced BODIPY-C11 mean fluorescence intensity (MFI) in N27 cells treated with OGD for 2 h. Data are means ± SEM, *n* = 6 wells from one representative of 3 independent experiments. *t* test was performed. **l** Cell viability of N27 cells 24 h after OGD and Liproxstatin-1 (Lip-1) of concentration gradient co-treatment. Data are means ± SEM, *n* = 6 wells from one representative of 3 independent experiments

To confirm the occurrence of ferroptosis in ischemic stroke, and to dissect out the necessary conditions triggering ferroptosis, we modulated the conditions of MCAO/R and studied the biochemical and behavioral changes. After MCAO/R in both mice and rats (24 h post reperfusion), the levels of lipid peroxides (LPO) and malondialdehyde (MDA) (a decomposition product of lipid peroxides) were significantly elevated in the affected hippocampal and cortical regions of the ipsilateral “stroke” hemisphere compared to the contralateral “control” hemisphere (mice: Fig. 4a, b; rats: Supplementary Fig. S2a, b). Transmission electron microscopy (TEM) images revealed that these features in the ipsilateral cortical regions 24 h post-MCAO/R were accompanied by neuronal mitochondrial shrinkage, characteristics of ferroptosis^22^ (Supplementary Fig. S2c).

Increasing ferroptotic proclivity by sub-toxic doses (where no neuronal loss was evident) of RSL3 (GPx4 inhibitor) and erastin (Xc^−^ inhibitor), delivered immediately after MCAO/R, markedly aggravated cerebral I/R injury in mice, evidenced by significantly elevated MDA levels (Fig. 4c and Supplementary Fig. S2d), neurological behavioral deficits (Fig. 4d and Supplementary Fig. S2e), and increased infarction volume (24 h post MCAO/R; Fig. 4e and Supplementary Fig. S2f). The lipid-radical trapping agents and archetypal ferroptosis inhibitors liproxstatin-1 and ferrostatin-1 have been shown previously to potently attenuate cerebral I/R injuries in the MCAO mouse model.^16^ However, these inhibitors could not rescue neuronal damage resulting from permanent MCAO (i.e., without reperfusion) (Fig. 4f, g) since ferroptosis is dependent on oxygen, further indicating that ferroptosis may occur during the reperfusion stage.

We corroborated the occurrence of neuronal ferroptosis in the oxygen-glucose deprivation (OGD) cell culture model of I/R. Death of N27 neuronal cells subjected to OGD for 2 h (Fig. 4h) was preceded by raised intracellular Fe^2+^ level (Fig. 4i), lipid peroxides (assayed by BODIPY 581/591 C11 [BODIPY]; Fig. 4j, k), lipid hydroperoxides (Liperfluo probe, Supplementary Fig. S2g, h), and total intracellular ROS and MDA (Supplementary Fig. S2i, j). The mitochondria in N27 cells treated by OGD were also significantly smaller, but with increased membrane density, compared to the control cells (Supplementary Fig. S2k). Liproxstatin-1 treatment rescued the OGD-induced neuronal death (Fig. 1l), showing the involvement of ferroptosis.

### Platelet activation after ischemic stroke

**Fig. 5:**
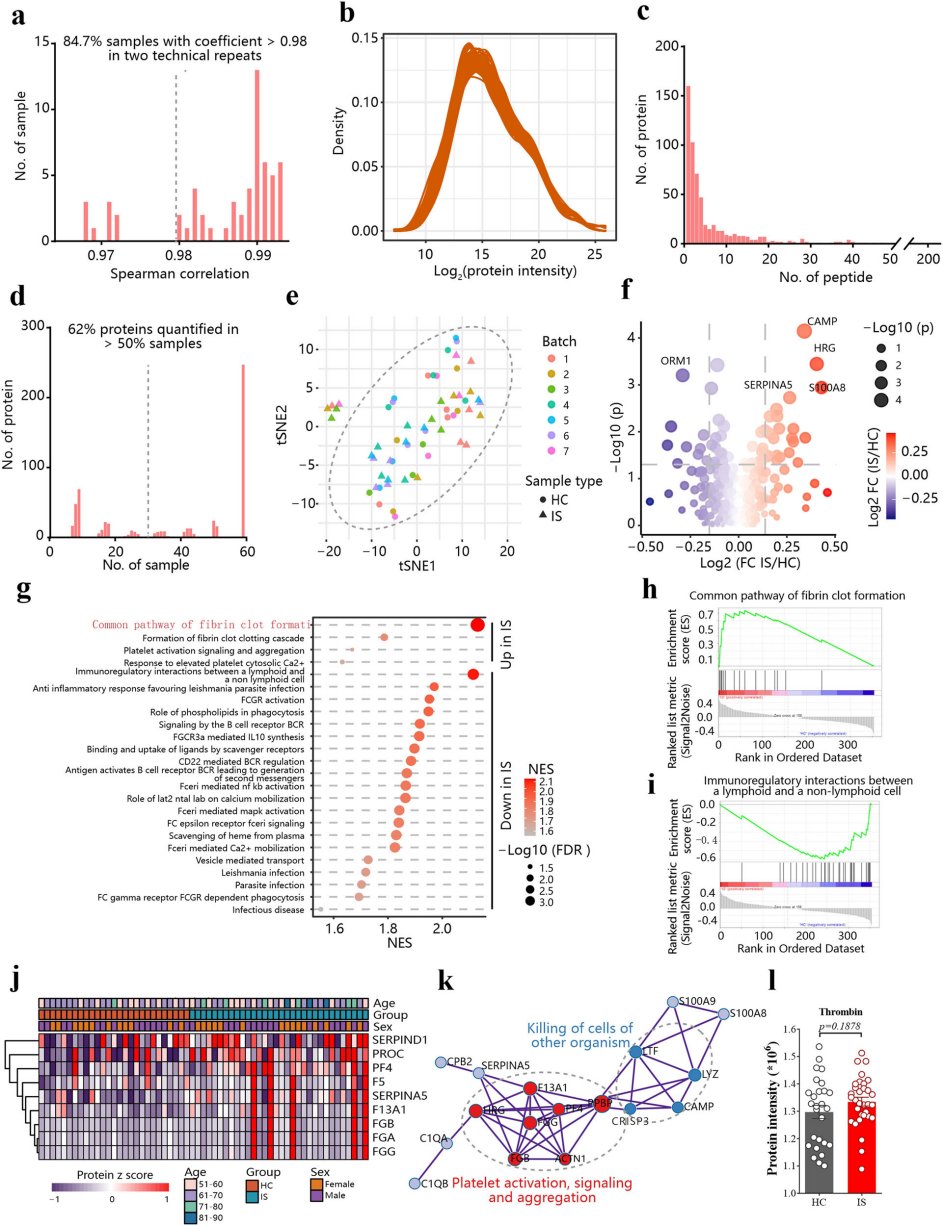
Platelet activation after ischemic stroke. **a** The statistic of Spearman coefficients for 59 samples in two technical workflow replicates. **b** The unimodal distributions of the protein intensity suggest no palpable degradation in the serum sample. **c** The distribution of peptides for quantified proteins. **d** The distribution of quantified protein numbers in samples. **e** tSNE analysis of the proteomics for 59 serum samples, and no batch effects were observed. healthy controls (HC), ischemic stroke (IS). **f** A volcano plot of the 362 proteins, quantified in > 50% samples, and imputed by random forest. **g** The significantly changed (*p* < 0.05 and FDR < 0.25) pathway in ischemic stroke patients, based on gene set enrichment analysis (GSEA). **h**, **i** GSEA analysis of common pathway of fibrin clot formation (**h**) and immunoregulatory interactions between a lymphoid and a non-lymphoid cell (**i**) gene sets enriched among 362 proteins. **j** A heatmap of GSEA enriched proteins in the common pathway of fibrin clot formation gene set from 362 proteins. **k** Protein–protein analysis of the 22 upregulated proteins in stroke. **l** Using mass spectrometry to detect plasma prothrombin abundance in clinical ischemic stroke (IS) patients and healthy controls (HC). Data are means ± SEM. HC, *n* = 27; IS, *n* = 32. *t* test was performed

The activation of coagulation function is the main cause of neuronal damage in ischemic stroke.^29^ Therefore, thrombin inhibition could potentially prevent and treat cerebral infarction, and reduce the magnitude of residual neurologic deficit following stroke.^30^ Clinical trials of thrombin inhibitors have been conducted based on the anticoagulant effect.^31^ However, we have found that thrombin in neurons can also trigger ferroptosis through its serine protease activity. The source of such thrombin elevation would be of interest to investigate. To study the changes in the coagulation system during ischemic stroke, we collected 59 serum samples, including 27 healthy controls and 32 ischemic stroke cases, and proceeded with TMT-labeled proteomics analysis after removing the high-abundant proteins in the serum (Fig. 5a–e). The correlation values of 84.7% of samples were more than 0.98, with a range from 0.968 to 0.993 (Fig. 5a), indicating that the results are reproducible. In total, we were able to identify 584 proteins in the analysis, in which 424 proteins could be supported by ≥2 peptides, with an average number of 8.31 (Fig. 5c). The distribution of proteins across diverse samples was evaluated, and we found that 362 proteins were quantified in more than 50% of samples (Fig. 5d). tSNE analysis, based on the Random Forest imputed data including 362 proteins, validated that no batch effect remained (Fig. 5e).

We identified in total 28 proteins upregulated in ischemic stroke serum, with 21 proteins downregulated (Fig. 5f). Among these, fibrin clot formation was significantly upregulated in the serum from ischemic stroke patients (Fig. 5g–k), which is consistent with the increased platelet aggregation in the mouse brain (Fig. 2f). However, there was no significant increase in thrombin levels in the serum compared to healthy controls (Fig. 5l), indicating the elevation we observed in the brain (Fig. 2h) may not come from the blood. Therefore, if dabigatran confers neuroprotection after ischemic stroke (Supplementary Fig. S2c, d), its beneficial action is unlikely to result from its activity in the blood to inhibit coagulation. Instead, the lack of elevation of thrombin in the blood of patients with ischemic stroke suggests that any benefit of anti-thrombin drugs is likely to be due to the elevation of thrombin in the brain (Fig. 2h). Given the additional role of thrombin to cause mobilization of the ferroptotic fatty acid, AA, we hypothesized that thrombin might induce ferroptosis after ischemic stroke.

### ACSL4 mediates thrombin neurotoxicity

**Fig. 6:**
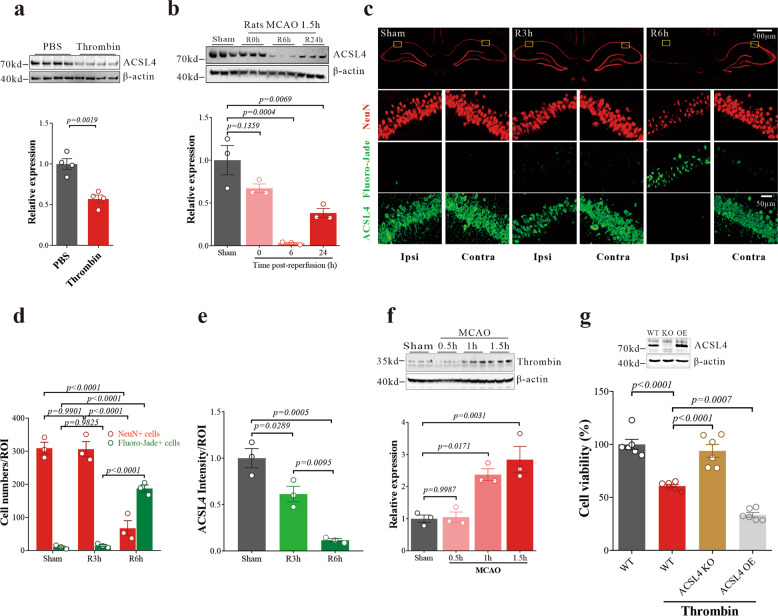
Thrombin induces the downregulation of ACSL4 after acute cerebral ischemia/reperfusion. **a** ACSL4 protein level was examined in N27 cells treated with thrombin (0.5 U/mL) for 24 h. Western blots were analyzed with Image J and normalized to β-actin expression. Data are means ± SEM, *n* = 4. *t* test was performed. **b** ACSL4 protein levels were examined from the ischemic ipsilateral hippocampus of rats that underwent MCAO after 0, 6, or 24 h of reperfusion. Western blots were analyzed with Image J and normalized to β-actin expression. Data are means ± SEM, *n* = 3 animals per group. One-way ANOVA with post-hoc Tukey test was performed. **c** Degeneration of CA1 pyramidal neurons following I/R. Hippocampal sections from sham, R3h (3 h after MCAO/R), and R6h (6 h after MCAO/R) rats were stained with NeuN (red) and Fluoro-Jade (green). Representative Immunofluorescence staining for ACSL4 from adjacent brain tissue sections spaced 4 μm apart. **d** Quantification of survival and death neuron numbers in a region of interest (ROI = 0.1 mm^2^) Data are means ± SEM, *n* = 3. Two-way ANOVA with post-hoc Tukey test was performed. **e** The intensity of ACSL4 immunofluorescence in an ROI was quantified using Image J. Data are means ± SEM, *n* = 3 animals per group. One-way ANOVA with post-hoc Tukey test was performed. **f** Thrombin protein levels were examined from the ischemic ipsilateral hippocampus of rats 0.5, 1, and 1.5 h after MCAO. Western blots were analyzed with Image J and normalized to β-actin expression. Data are means ± SEM, *n* = 3 animals per group. One-way ANOVA with post-hoc Tukey test was performed. **g** Cell viability of WT, ACSL4 KO, and ACSL4 OE N27 cells 24 h after thrombin (0.5 U/mL) treatment. Data are means ± SEM, *n* = 6 wells from one representative of 3 independent experiments. One-way ANOVA with post-hoc Tukey test was performed

Acyl‐CoA synthetase long‐chain family member 4 (ACSL4), an important enzyme involved in lipid metabolism, participates in ferroptosis by converting free AA into arachidonoyl‐CoA^11^ to generate lipid hydroperoxides. Consistent with free AA suppressing ACSL4 levels by promoting its ubiquitination and proteasomal degradation,^32^ we found that ACSL4 was decreased in N27 cells after thrombin treatment (Fig. 6a), without affecting *Acsl4* mRNA expression (Supplementary Fig. S3a). The protein expression of ACSL4 in the hippocampus of the ischemic region decreased significantly at 6 h after I/R (Supplementary Fig. S3b), while the expression of *Acsl4* mRNA did not change significantly at the same time point (Supplementary Fig. S3c). Since the free AA in the hippocampus of mice increased significantly after ischemic stroke (Fig. 1k), these data further indicate that the decrease of ACSL4 during I/R may be the result of a post-translational modification. We also found that the hippocampal ACSL4 expression decreased similarly over time in the ipsilateral side of rats post MCAO/R, as assayed by western blot (Fig. 6b) or histology (Fig. 6c). The decrease in ACLS4 was apparent at 3 h post-I/R, which preceded hippocampal neuronal loss (assessed using NeuN for surviving neurons and Fluoro-Jade for degenerating neurons) commencing from 6 h post-I/R (Fig. 6d, e). The results in rats were consistent with results in mice after MCAO (Supplementary Fig. S3d–f). However, thrombin was significantly elevated in the early stage of ischemia (Fig. 6f). These data indicate that the downregulation of ACSL4 was an early event during cerebral I/R, and occurred independently of neuronal death, which could be a protective response to thrombin-induced stress.

To clarify whether thrombin cytotoxicity is mediated by ACSL4, we have generated *Acsl4* knockout (KO) N27 cells using CRISPR-Cas9-based gene editing, and *Acsl4* over-expressing (OE) cells using a lentiviral vector pLenti-OE-rACSL4 containing an *Acsl4* expression cassette. Both modulations were confirmed by western blotting (Fig. 6g, top panel). We found that thrombin is dependent on ACSL4 to cause toxicity since cytotoxicity of thrombin was rescued by *Acsl4* reduction and aggravated by *Acsl4* overexpression (Fig. 6g). The ACSL4 inhibitor pioglitazone (PIO) also blocked the cytotoxicity of thrombin (Supplementary Fig. S3g). These results indicate that thrombin may contribute to neuronal cell death by promoting ACSL4-dependent ferroptosis, and that the decrease of ACSL4 may be beneficial against ferroptotic damage induced by thrombin.

### Modulating ACSL4 expression alters outcomes of acute ischemic brain injury

**Fig. 7:**
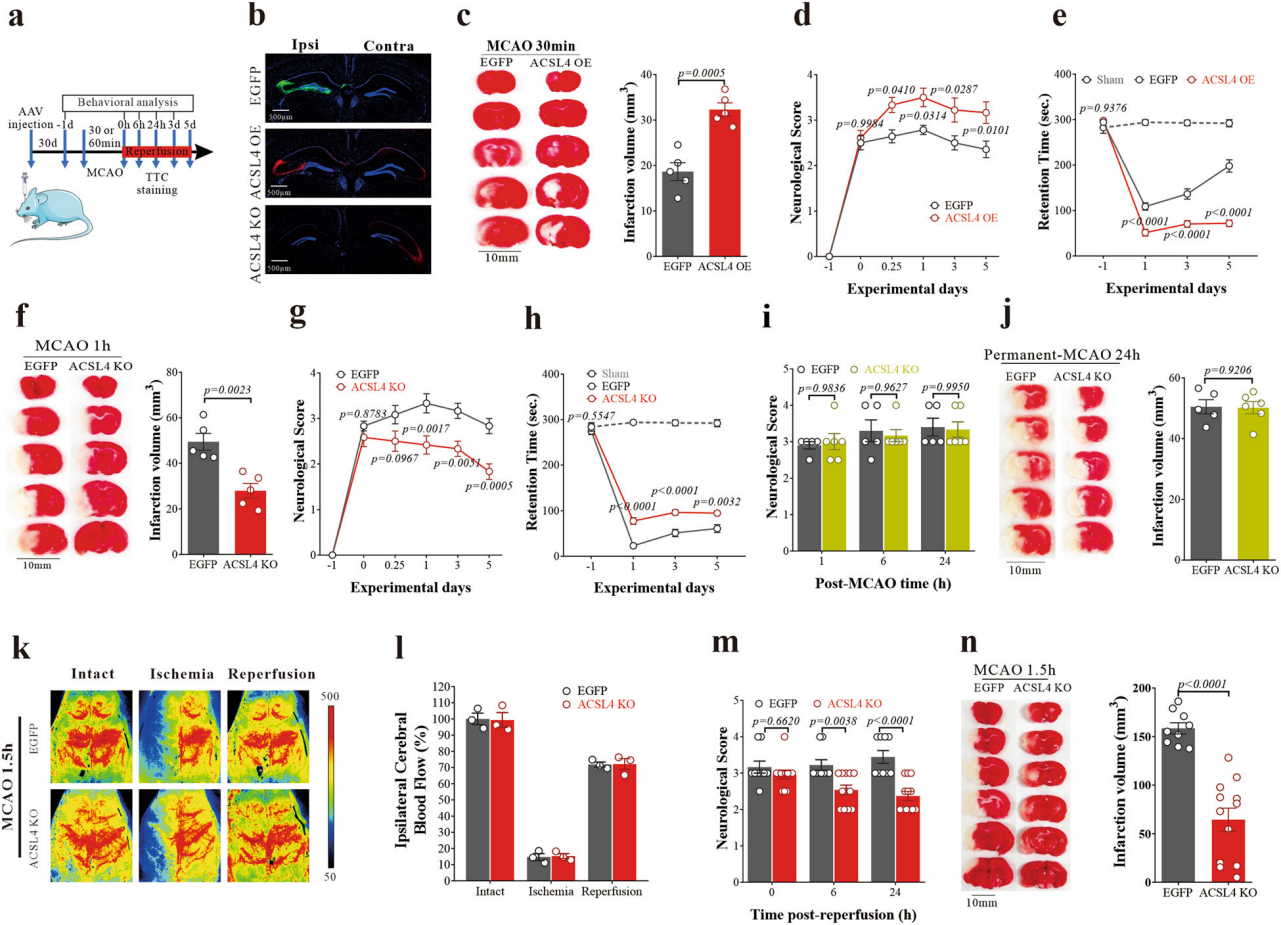
Modulating ACSL4 expression altered the outcomes of acute ischemic brain injury. **a** Schematic of the experimental paradigm. **b** The location of AAV injection and ACSL4 expression in the brain of mice were verified. **c** Representative images of TTC staining of EGFP and ACSL4 OE mice 24 h after 30 min of MCAO. Quantification of infarction volume indicated by TTC staining using Image J. Data are means ± SEM, *n* = 5 animals per group. *t* test was performed. **d** The neurological score was performed at 0 h, 6 h, 24 h, 3 days, and 5 days after MCAO/R. Data are means ± SEM. EGFP, *n* = 7; ACSL4 OE, *n* = 9. Two-way ANOVA with post-hoc Sidak test was performed. **e** The performance on the rotarod test was analyzed at 1 day, 3 days, and 5 days after MCAO surgery. Data are means ± SEM. Sham, *n* = 6; EGFP, *n* = 7; ACSL4 OE, *n* = 9. Two-way ANOVA with post-hoc Tukey test was performed. **f** Representative images of TTC staining of EGFP and ACSL4 KO mice 24 h after 60 min of MCAO. Quantification of infarction volume indicated by TTC staining using Image J. Data are means ± SEM, *n* = 5 animals per group. *t* test was performed. **g** Neurological scoring was performed at 0 h, 6 h, 24 h, 3 days, and 5 days after MCAO/R. Data are means ± SEM, *n* = 6 animals per group. Two-way ANOVA with post-hoc Sidak test was performed. **h** The performance on the rotarod test was analyzed at 1 day, 3 days, and 5 days after MCAO surgery. Data are means ± SEM, *n* = 6 animals per group. Two-way ANOVA with post-hoc Tukey test was performed. **i** Neurological scoring was performed at 1, 6, and 24 h after permanent-MCAO. Data are means ± SEM. EGFP, *n* = 5; ACSL4 KO, *n* = 6. Two-way ANOVA with post-hoc Sidak test was performed. **j** Representative images of TTC staining of EGFP and ACSL4 KO mice 24 h after permanent-MCAO. Quantification of infarction volume indicated by TTC staining using Image J. Data are means ± SEM. EGFP, *n* = 5; ACSL4 KO, *n* = 6. *t* test was performed. **k** Representative images obtained from PeriCam PSI System. The brighter area indicates higher blood perfusion. **l** Cortical blood flow changes before and after MCAO/R in rats. **m** The neurological score was performed at 0, 6, and 24 h after MCAO/R in rats. Data are means ± SEM. EGFP, *n* = 9; ACSL4 KO, *n* = 12. Two-way ANOVA with post-hoc Sidak test was performed. **n** Representative TTC-stained serial brain sections of rats 24 h after MCAO/R, where viable tissue stains red. Quantification of infarction volume indicated by TTC staining using Image J. Data are means ± SEM. EGFP, *n* = 9; ACSL4 KO, *n* = 12. *t* test was performed

To determine whether ACSL4 impacts on outcomes after cerebral I/R, we selectively overexpressed or knocked out *Acsl4* in the mouse left hippocampal CA3 region (the most vulnerable region to MCAO/R in mice) using single injections of adeno-associated viral vectors, namely, AAV8-mACSL4 (overexpression, OE) and AAV8-EF-Cas9+AAV8-mACSL4-sp.g3 (knockout, KO), and examined the effects in the MCAO rodent models (Fig. 7a). AAV8-EGFP (AAV8 with enhanced green fluorescent protein) was used as a control. The effectiveness of transduction was confirmed by immunofluorescence staining (Fig. 7b). Subjected to the same 30 min MCAO/R, ACSL4 OE mice exhibited significantly increased infarct volume (24 h post-perfusion, Fig. 7c), worsened neurological scores (Fig. 7d), and poorer rotarod motor performance (Fig. 7e) up to 5 days post-reperfusion compared to EGFP mice. In contrast, ACSL4 KO mice were protected against I/R injuries, evidenced by reduced infarct volume (Fig. 7f), as well as significantly improved neurological scores (Fig. 7g) and motor coordination (Fig. 7h) compared to the EGFP mice. Consistent with ferroptosis inhibitors, there was no difference in neuronal damage after permanent MCAO in ACSL4 KO and control mice (Fig. 7i, j), suggesting strongly that ferroptosis occurred during reperfusion in this model.

These results in mice were replicated in rats since one major issue for experimental stroke research is the reproducibility of animal models.^33,34^ Laser speckle signals indicated that knockout of ACSL4 did not affect the blood flow in the rat cortex after MCAO/R (Fig. 7k, l), confirming that ACSL4 KO was not protective simply by affecting hemodynamics. AAV-assisted ACSL4 knockout in rat cortex conferred marked protection against I/R induced functional impairments (neurological score; Fig. 7m) and brain infarct volume (TTC staining; Fig. 7n).

We have found that ACSL4 KO cells were protected against ferroptosis induced by RSL3, whereas ACSL4 OE cells were made vulnerable to the same toxin (Supplementary Fig. S4a). We then subjected these cells to OGD/reoxygenation and found that ACSL4 KO cells were resistant to, and ACSL4 OE cells were more vulnerable to OGD-related toxicity (Supplementary Fig. S4b). Pharmacological inhibition of ACSL4 using triacsin C,^35^ or PIO,^36^ prevented I/R injury, as evidenced by significantly improved neurological scores (triacsin C: Supplementary Fig. S4c; PIO: Supplementary Fig. S4e) and reduced infarct volume 24 h post-reperfusion (triacsin C: Supplementary Fig. S4d; PIO: Supplementary Fig. S4f). PIO was also found to protect against OGD in vitro in normal N27 cells (Supplementary Fig. S4g) but not ACSL4 KO cells (Supplementary Fig. S4h).

## Discussion

**Fig. 8:**
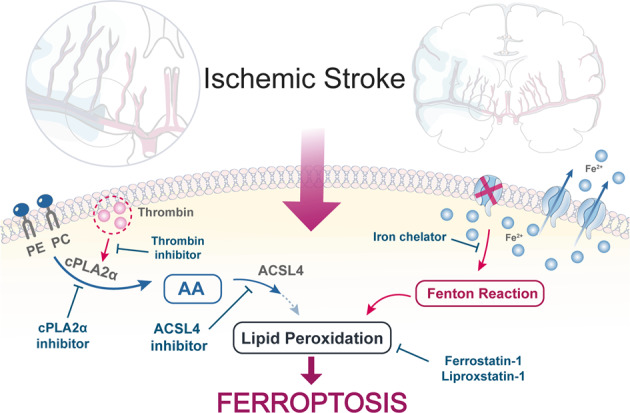
Schematic hypothesis. Cerebral ischemia leads to an unexpected increase in thrombin within neurons, promotes the mobilization of PE and PC in the phospholipid membrane of neuronal cells through cPLA2α, and accelerates the production of AA. Under the catalytic action of ACSL4, AA is esterified and made available as a ferroptotic fuel. This injury process can be blocked by thrombin inhibitors, cPLA2α inhibitors, and ACSL4 inhibitors. Concurrently, iron accumulates during I/R, which also contributes to ferroptosis and can be blocked by an iron chelator

Here we found that the thrombin–ACSL4 axis promotes ferroptotic cell death after ischemic stroke, and pharmacologically inhibiting this pathway may provide a new avenue for therapy. The mechanisms for how neurons die after ischemic stroke are not well understood, and these findings build upon prior results demonstrating the importance of ferroptosis in ischemic disease.^16,18^ Using proteomics, lipid metabolomics, and immunochemistry, we identified thrombin and its downstream partner in AA metabolism, ACSL4, as critical proteins involved in I/R-related ferroptotic cell death, and thus provide a new understanding of the biochemistry driving ferroptosis in ischemic stroke (Fig. 8).

Thrombin is a known drug target for ischemic stroke, but our findings illuminate machinery beyond coagulation and, for the first time, implicate thrombin in ferroptosis. While historically only thought to be involved in blood clotting, additional roles for thrombin were predicted when the cellular receptor for thrombin, the protease-activated receptors (PARs), were identified.^37^ Thrombin receptor knockout mice were resistant to I/R-induced cerebral injury,^38^ and thrombin inhibitors have been shown to reverse ischemic brain injury.^39^ We have shown that the cytotoxic effect of thrombin is dependent on the expression of the ferroptotic gene, *Acsl4*. More importantly, dabigatran and argatroban, two thrombin inhibitors, have entered Phase III (NCT03961334) and Phase IV (NCT03740958) clinical trials, respectively. These drugs inhibit blood coagulation, and it is thought that this mechanism acts to promote blood flow after ischemic stroke. Yet, given the MCAO model does not involve blood clotting as a primary mechanism (blood flow is occluded mechanically, not by a clot^34^), it is unlikely that the benefits of thrombin inhibitors reported for this model are mediated by fibrinolysis or clot dispersion. Instead, ferroptosis may be the target of these drug candidates.

Thrombin, a Na^+^-activated serine protease, is expressed in an inactive form, prothrombin, which combines with Na^+^ to become active thrombin in the brain. Prothrombin and thrombin have been localized to neurons and glial cells in the CNS,^40^ and the prothrombin mRNA has been shown to be elevated in the hippocampus in a rodent model of cerebral ischemica.^41^ In the present study, we detected thrombin protein in the nonischemic brain (Fig. 6f), consistent with thrombin being a resident brain protein. But prothrombin may also enter the brain from the blood when the blood-brain barrier is disrupted.^42^ Indeed, in rodent models, areas of the brain with severe vascular damage resulting from ischemia were shown to have increased thrombin compared to regions that were relatively spared.^43^ with most (65%) thrombin co-localized in neurons, compared with microvessels (15%), and glial cells (10%). However, with our observations that thrombin was not significantly changed in the serum of ischemic stroke patients (Fig. 5l), it is likely that brain-derived thrombin participates in neuronal ferroptosis.

Thrombin activates cPLA2α by increasing cytosolic Ca^2+^ and also by phosphorylating cPLA2α, which causes AA to be released.^27,44–49^ cPLA2α phospholipid hydrolysis that releases PUFA stimulates the conventional lipoxygenase biosynthesis of lipid mediators.^50^ There are two important members of the phospholipase A2 superfamily that display a proclivity toward plasmalogen phospholipids with arachidonate at the sn-2 position: cPLA2α and iPLA2β.^51^ cPLA2α, which is a calcium-dependent phospholipase, cleaves AA at the sn-2 position of glycerophospholipids to release AA from the membrane,^52^ and promotes ferroptosis through the accumulation of iron-dependent lethal lipid peroxides.^53^ Through these actions of the AA cascade, cPLA2α has been implicated in the pathophysiology of inflammation-related diseases and cancer.^54^ However, iPLA2β, which is calcium-independent, cleaves acyl tails from the glycerol backbone of lipids, which causes oxidized fatty acids from phospholipids to be released.^55^ This action suppresses ferroptosis by depleting oxidized lipids.^56^ Indeed, in xenograft mouse models, inhibition of iPLA2β increases sensitivity toward p53-driven ferroptosis resulting in tumor supression.^55^ Dependent on the oxidative status of the AA, cPLA2α or iPLA2β impacts the execution of ferroptosis sequentially, which may have implications in ischemic stroke.

Consistent with PLA2 contributing to ferroptotic stress during stroke, elevated lipoprotein-associated PLA2 (Lp-PLA2) activity in the acute period of ischemic stroke predicted near-term risk of recurrent vascular events,^57–60^ and risk of all-cause mortality in the ensuring year^61^ in clinical cohort studies. cPLA2α expression and activity are likewise increased in cerebral ischemia in animal studies, and cPLA2α inhibitor treatment or knockouts of PLA2 significantly reduced infarction volume following cerebral ischemia.^62–67^ Therefore, inhibitors of cPLA2α may also warrant further clinical tests similar to ferroptosis inhibitors.

As discussed above, thrombin partners with cPLA2α to release AA from the membrane,^44^ and ACSL4 catalyzes the esterification of AA into PE.^36^ AA-PE is the primary substrate for iron-induced peroxidation in ferroptosis.^68^ The loss of ACSL4 during ischemic stroke as we reported here (Fig. 6b), therefore, may be a neuroprotective response that we show can be amplified if ACSL4 is suppressed or inhibited (Fig. 7f–h, m, n and Supplementary Fig. S4c–f). The decrease in ACSL4, limiting AA-PE, is beneficial in an infarct penumbra where significantly elevated thrombin can activate cPLA2α.

In recent years, the effects of thrombin were investigated in a larger scope, and it may have functions in neuronal signaling, development, and plasticity.^69^ With the increasing awareness of the importance of thrombin in maintaining CNS function, studies have implicated it in various neurological diseases.^69^ Elevated thrombin is implicated in neurodegenerative diseases, including Alzheimer’s disease,^70^ Parkinson’s disease,^71^ multiple sclerosis,^72,73^ as well as traumatic^74^ and hemorrhagic brain injury.^75^ Coincidentally, ferroptosis have been implicated in those diseases as well.^20,76–83^ It is worth further investigation to link these abnormal features of diseases. In addition, ferroptosis have also been implicated in neoplastic diseases^84,85^ and visceral injuries (such as heart,^86–89^ liver,^90,91^ lung,^92^ kidney,^93–95^ and intestine^96,97^). The role of thrombin in these diseases may be of interest to investigate.

In summary, our findings strongly argue that anti-thrombin therapeutics may be beneficial post-reperfusion in stroke via ferroptosis inhibition, and may also be useful for other diseases where ferroptosis is implicated.

## Materials and methods

### Patient descriptions

Human blood sera were collected from healthy controls (*n* = 27) and ischemic stroke patients (*n* = 32). All participants enrolled in this study underwent a Montreal Cognitive Assessment (MoCA). Clinical information of all participants was recorded (Supplementary Table S1). The diagnosis of ischemic stroke was based on World Health Organization criteria^98^ and radiological findings from computed tomography or magnetic resonance imaging, reviewed by two independent neurologists. The study was approved by the Biomedical Research Ethics Committee and the Committee on Human Research of West China Hospital, Sichuan University (Reference No. 2016 [335]). Informed consent was obtained from participants or their guardians. After clotting and centrifugation, the sera were frozen and stored at −80 °C in aliquots of polyethylene tubes until use.

### Reagents

Reagents were purchased from Sigma-Aldrich unless specified.

### Cell lines and conditions

N27 cells, derived from E12 rat mesencephalic tissue (Merck, Bayswater, Australia), were cultured in RPMI 1640 (Gibco, Thermo Fisher Scientific) supplemented with 10% fetal bovine serum (Gibco, Thermo Fisher Scientific) in a 37 °C incubator with a humidified atmosphere of 5% CO_2_.

### OGD and reoxygenation

Oxygen-glucose deprivation and reoxygenation experiments were performed as previously described.^99^ Briefly, N27 cells were grown in complete media supplemented with glucose (4.5 g/L) for 24 h in normoxic conditions (5% CO_2_ and 21% O_2_). To initiate oxygen-glucose deprivation (OGD), N27 cells were exposed to deoxygenated glucose-free RPMI 1640 (Gibco, Thermo Fisher Scientific) in a humidified atmosphere containing 95% N_2_ and 5% CO_2_ at 37 °C for 2 h in an incubator (Serico CB, Binder GmBH, Tultingen, Germany). After 2 h challenge, cultures were removed from the anaerobic chamber, and the OGD solution in the cultures was replaced with a maintenance medium. Cells were then allowed to recover for 18 h in a regular incubator. Control cells were incubated for 20 h in 5% CO_2_ and 21% O_2_ in a media identical to the OGD media except for the addition of glucose.

### Cell viability assays

Cells were seeded onto 96-well plates (2000 cells per cell) and treated with the compounds [RSL3 (Selleck Chemicals), Erastin (Selleck Chemicals), Thrombin (Abcam), Liproxstatin-1 (Selleck Chemicals), Pioglitazone (Sigma-Aldrich), DMSO, Dabigatran (Selleck Chemicals), Trifluoroacetic acid (TFA, Sigma-Aldrich), Darapladib (Selleck Chemicals), NAC (Beyotime)] after plating. Cell viability was assessed at different time points after treatment (24 h unless stated otherwise) using Cell Counting Kit-8 (CCK-8) cytotoxicity assay (Bimake, B34304), as previously described.^13^ The cell death curve of RSL3 in N27 cells, as well as thrombin in MDA-MD-231 cells, are shown in Fig. S5.

### Assessment of lipid peroxidation with BODIPY and Liperfluo staining and flow cytometry

Lipid peroxidation within cells was assessed as previously described.^36^ In all, 160,000 cells per well were seeded in 6-well dished (Cell Ter) 1 day before the experiment. On the next day, cells were treated with OGD for 2 h. Cells were incubated with BODIPY 581/591 C11 (1 μM; Thermo Fisher) or Liperfluo (10 μM; Dojindo) for 30 min at 37 °C in a tissue culture incubator before harvest by trypsinization. Subsequently, cells were resuspended in 500 μL fresh PBS (DPBS, Gibco), strained through a 40 μM cell strainer (BD Falcon), and analyzed using the 488 nm laser of a flow cytometer (LSR Fortessa, BD) for excitation. For BODIPY 581/591 C11 staining, the signals from both non-oxidized C11 (PE channel) and oxidized C11 (FITC channel) were monitored. The ratio of mean fluorescence intensity (MFI) of FITC to MFI of PE was calculated for each sample. In other cases, only the signal from oxidized C11 was monitored, and the MFI of FITC was calculated. The data were normalized to control samples as shown by the relative lipid ROS. Data were collected from the FL1 detector (BODIPY/Liperfluo) with a 502 low-pass and 530/30 band-pass filter. At least 10,000 cells were analyzed per sample. Data analysis was conducted using the FlowJo Software.

### Animals

Adult male C57BL/6 mice (25–30 g) and Sprague-Dawley rats (250–300 g) were housed under standard conditions of temperature and humidity, and a 12 h light/dark cycle (lights on at 08:00), with free access to food and water before use. Adequate measures were taken to minimize pain or discomfort during surgeries. All mice and rat experiments were carried out following the Institutional Guidelines of the Animal Care and Use Committee (K2018071, Sichuan University, China). For all animal experiments, we used a randomized, double-blind experimental setting. Detailed steps include (1) Randomized grouping of experimental animals (experimenter I); (2) Injecting AAV or drug treatment, and numbering (experimenter II); (3) Constructing the MCAO model and behavioral assessment and TTC staining (experimenter III); (4) Quantification and statistical analysis (experimenter IV).

### Focal cerebral ischemia model

All surgeries were conducted under aseptic conditions by a skilled animal surgeon. Transient acute focal cerebral ischemia was induced by reversible intraluminal MCAO, as described previously.^16^ Male mice or rats were anesthetized with isoflurane (5% induction and 1% maintenance). A 2 cm incision was opened in the middle of the anterior neck. Left unilateral MCAO was accomplished by inserting a Silicon rubber-coated nylon monofilament (Guangzhou Jialing Biotechnology Co., Ltd., China) into the internal artery via the common carotid artery, advanced 9–10 mm (in mice) or 20–21 mm (in rats) past the carotid bifurcation until a slight resistance was felt. The adequacy of MCAO was confirmed by monitoring cortical blood flow using a PeriCam PSI System (Perimed, Järfälla, Sweden). Animals were excluded if the mean ipsilateral laser speckle signal was >30% pre-ischemic ipsilateral hemisphere baseline. Body temperature was controlled at 36.5 ± 0.5 °C throughout MCAO surgery with a heating pad. After 30 or 60 min (mice) or 90 min (rats) of occlusion, the occluding filament was withdrawn to allow for reperfusion, and the incision was closed with 4-0 surgical sutures (JINHUAN, Co., LTD.). In the sham-operated animals, the occluding filament was inserted only 5 mm above the carotid bifurcation. The surgeon was blinded to treatment groups.

### Adeno-associated viral vectors production and stereotaxic injection

To overexpress mouse ACSL4 by recombinant adeno-associated virus (rAAV) in vivo, ACSL4 cDNA of the mouse was introduced into AgeI and SacI cleaved plasmid pssAAV-CB-eGFP to generate pssAAV-mACSL4. The plasmids containing the selected sgRNAs for the knockout of ACSL4 pssAAV-mACSL4-sp.g3 (for mouse) and pX601-AAV-rACSL4-sa.g1 (for rat), the plasmid for mouse ACSL4 overexpression pssAAV-mACSL4, the plasmid for cas9 expression pssAAV-EF-cas9, and the plasmid for eGFP expression pssAAV-CB-eGFP were used to produce rAAV vector in serotype 8 (rAAV8). All the rAAV8 vectors were generated by a triple-plasmid cotransfection method in human embryonic kidney 293 cells. The rAAV8 vectors were collected at 72 h post-transfection and purified by two rounds of CsCl gradient ultracentrifugation, followed by silver staining and genome copy titration, as described previously.^100^ The viral vectors were aliquoted and stored at - 80 °C before use.

All surgeries were conducted under aseptic conditions. rAAV injections in specific brain regions were carried out with a stereotaxic instrument (World Precision Instruments). For each mouse, 2 μL of ssAAV8-mACSL4 (1 × 10^13^ GC/ml) or 1 μL of ssAAV8-mACSL4-sp.g3 (1 × 10^13^ GC/ml) and 1 μL of ssAAV8-EF-cas9 (1 × 10^13^ GC/ml) were injected into CA3 of the left hippocampus (Bregma: −2 mm, left lateral: 2 mm, depth: 2 mm); for each rat, 2 μL of AAV8-rACSL4-sa.g1 (1 × 10^13^ GC/ml) was injected into the left cerebral cortex (Bregma: −4.3 mm, left lateral: 4 mm, depth: 2 mm). By contrast, control mice or rats received 2 μL injections of ssAAV8-CB-eGFP (1 × 10^13^ GC/ml). The needle was left in place for 5 min after the injection was completed and withdrawn at a rate of 1 mm/min. Proper postoperative care was taken until the animals recovered completely. At 30 days after injection, focal cerebral ischemia was induced in mice and rats by MCAO and reperfusion, and neurological assessment and infarct volume measurement were performed at 24 h post-reperfusion unless stated elsewhere.

### Drug treatment

The mice or rats were chosen randomly for treatment (by Excel 2016). RSL3 (30 mg/kg, S8155, Selleck Chemicals), Erastin (10 mg/kg, S7242, Selleck Chemicals), or vehicle (2% DMSO) were delivered intranasally by pipette to the C57BL/6 mice immediately after MCAO/R. Ferrostatin-1 (10 mg/kg, S7243, Selleck Chemicals), Liproxstatin-1 (10 mg/kg, S7699, Selleck Chemicals), or vehicle (2% DMSO) were delivered intranasally by pipette to the C57BL/6 mice immediately after MCAO/R. Pioglitazone (1 mg/kg/day, IP, CDS021593, Sigma-Aldrich), Triacsin C (4 mg/kg/day, gavage, 10007448, Cayman Chemical) or vehicle (10% DMSO) was administered for 5 days before the induction of focal ischemia in C57BL/6 mice. Dabigatran (5 mg/kg, S2196, Selleck Chemicals) or vehicle (10% TFA water solution) was injected into the SD rats at 1 h before surgery via the caudal vein.

### Neurological assessment

The neurological assessment post-surgery was performed by an investigator blinded to the experimental groups and confirmed by a second investigator blinded to the experimental groups. After 0, 6, and 24 h of MCAO/R, the neurological deficit of each mouse or rat was evaluated by a five-point scale as described previously^16^: 0, no observable deficit; 1, right forelimb flexion; 2, decreased resistance to left lateral push (and right forelimb flexion) without circling; 3, same behavior as grade 2, with circling to the right; 4, severe rotation progressing into barreling, loss of walking or righting reflex.

### Rotarod treadmill test

Motor coordination of the animals after operation and treatment was measured using a rotarod treadmill for mice (SANS) under the accelerating rotor mode (10 speeds from 4 to 40 r.p.m. for 5 min), as previously described.^16^ The interval from when the animal mounted the rod to when it fell off was recorded as the retention time, and mice that lasted for 300 s on the accelerating rotating rod were recorded as survivors.^16^ The animals were trained for 2 days, 3 trials per day, before surgery, and the mean duration on the rod was recorded to obtain stable baseline values. Performance on the rotarod test was measured 3 times a day in the 5 days following the ischemic insult.

### Infarct volume analysis

The individual performing the infarct volume analysis was blinded to the treatment group. At 24 h of reperfusion, the mice or rats were euthanized, and brains were removed rapidly and placed at −20 °C for 20 min. Coronal slices were made at 2 mm intervals from the frontal poles, and the 2-mm brain sections were incubated in 1% 2,3,5-triphenyl tetrazolium chloride (TTC, Sigma-Aldrich) in phosphate-buffered saline (PBS) for 15 min at 37 °C, and then fixed in 10% formalin for 24 h. Infarction volume was measured using digital imaging as previously described,^16^ and images were analyzed using Image J (1.49 m, NIH) by an investigator blinded to the experimental groups. The area of infarct (white, unstained), the area of the ipsilateral hemisphere (white, unstained, plus red brick, stained), and the area of the contralateral hemisphere (red brick, stained) were measured for each section by a blinded operator. The volume was calculated by summing the representative areas in all sections and multiplying by the slice thickness, then correcting for edema, as previously described^16^: Corrected Infarct Volume (CIV) = contralateral hemisphere volume – (ipsilateral hemisphere volume − infarct volume).

### Detection of arachidonic acid in mice

Mice were deeply anesthetized using chloral hydrate (BBI Life Sciences) and transcardially perfused with PBS before the brains were removed. Mouse arachidonic acid (AA) ELISA Kit (JL13827, Jianglai, China) was used to detect the free AA level in lysates of brain tissue, following the manufacturer’s protocols. The cerebral cortex and hippocampus were dissected and removed residual blood by washing tissue with pre-cooling PBS buffer (0.01 M, pH = 7.4). Mince tissue after weighing it and get it homogenized in PBS (the volume depends on the weight of the tissue) with a glass homogenizer on ice and broke by an ultrasonic cell disrupter. The homogenates are then centrifuged for 5 min at 5000 × *g* to get the supernatant. Add 50 μL standard or sample into each microtiter well pre-coated with anti-AA antibody (MBS2003715, MyBioSource). Immediately add 100 μL HRP-labeled antibody into each well, gently tap the plate to ensure thorough mixing, then incubate for 60 min at 37 °C. In all, 100 μL of Chromogen Solutions were added to each well and incubated for 15 min at 37 °C. Add 50 μL Stop Solution. Read at 450 nm immediately and calculate. All procedures were performed according to the manufacturer’s protocol.

### Cell death measurements

Neuronal loss was quantified as previously described.^101^ Briefly, 3 and 6 h after I/R (R3h, R6h), animals were deeply anesthetized and transcardially perfused with 0.9% NaCl saline followed by ice-cold 4% paraformaldehyde in 0.1 M phosphate-buffered saline (PBS). Brain sections (4 µm) were processed for NeuN staining with mouse anti-NeuN (1:1000; Servicebio, GB11138) and reacted with conjugate-absorbed goat anti-rabbit Cy-3 (1:300, Servicebio, GB21303) to determine surviving neurons. The sections were dried overnight for Fluoro-Jade (Sigma-Aldrich), which selectively stains degenerating neurons. The slides were immersed for 3 min in 100% ethanol, for 1 min in 70% ethanol, for 1 min in distilled water, and then transferred to a solution containing 0.01% Fluoro-Jade and 0.1% acetic acid (1:10) for 30 min on a shaker. After three 10-min washes, the slides were finally coverslipped. Labeled sections were imaged with a confocal laser-scanning microscope (Nikon ECLIPSE Ti-S). The number of cells in each section was divided by the area sampled, which was determined using imaging probes.

### Immunofluorescence

For immunofluorescence of brain tissue sections, the brain was isolated and fixed by 4% paraformaldehyde in PBS overnight and then cut into 4 µm paraffin sections. After staining with primary antibody (ACSL4, Abcam, ab155282; NeuN, Servicebio, GB11138; EGFP, Servicebio, GB11602) and fluorescent-tagged secondary antibody, nuclei were counterstained with 4,6-diamidino-2-phenylindole (DAPI), and coverslips were placed. Labeled sections were imaged with a confocal laser-scanning microscope (Nikon ECLIPSE Ti-S).

### LC-MS/MS analysis of proteomics

The desalted peptides were dried in a speed vacuum and resuspended in buffer A (2% ACN, 0.1% FA), LC-MS/MS analysis was performed using an EASY-nanoLC 1000 nanoflow LC instrument coupled to a high-resolution mass spectrometer (Q Exactive Plus, Thermo Fisher Scientific). A 100 μm (inner diameter) × 2 cm (length) of trap column and a 75 μm (inner diameter) × 12 cm (length) of the analytical column was in-house pulled and packed with C18 particle (DIKMA). Data-dependent acquisition (DDA) was performed in positive ion mode at the flow rate of 300 nL/min. MS spectra were acquired from 350 *m*/*z* to 1600 *m*/*z* with a resolution of 70,000 at *m*/*z* = 200. The automatic gain control (AGC) value was set at 3e6, with a maximum injection time of 20 ms. For MS/MS scans, the top 15 most intense parent ions were selected with a 0.6 *m*/*z* isolation window and fragmented with normalized collision energy (NCE) of 30%. The AGC value for MS/MS was set to a target value of 1e5, with a maximum injection time of 100 ms and a resolution of 35,000. Parent ions with a charge state of *z* = 1 or with unassigned charge states were excluded from fragmentation, and the intensity threshold for selection was set to 2e5.

### Data searching for proteomics

All the raw files were searched against the Swiss-Prot human protein sequence database (20413 entries, 2017/01/14) in Maxquant (version 1.6). The precursor peptide mass tolerance was 10 ppm, and the fragment ion mass tolerance was 0.02 Da. Two missed trypsin cleavages were allowed. Cysteine carbamidomethylation was set as a fixed modification. Oxidation of methionine and protein N-terminal acetylation were set as variable modifications. Peptides with <1% false discovery rate (FDR) were chosen for further data processing.

### Data processing for proteomics

We selected the proteins that have at least two peptides, and then the intensity of each protein was log2 transformed. The *p* values calculated by the linear model and Bayes moderated *t*-statistics packaged in R/Bioconductor software of Limma were applied to assess the differentially expressed proteins in the “stroke” group compared with the “control” group.

### LC-MS/MS analysis of lipidomics

The lipidomics was analyzed in Shanghai Applied Protein Technology Co., Ltd. Specifically, the temperature of the automatic sampler was set to 10 °C. In all, 2 μL of the sample was injected onto a reverse-phase CSH C18 column (Waters, ACQUITY UPLC CSH C18, 1.7 μm, 2.1 mm × 100 mm) using a UPLC system (Nexera LC-30A, SHIMADZU). Analyses were performed under the following conditions: column temperature 45 °C; the mobile phase A: 10 mM of ammonium formate in aqueous acetonitrile (acetonitrile: water = 6:4, v/v); the mobile phase B: 10 mM of ammonium formate in a mixed solvent of isopropanol and acetonitrile (acetonitrile: isopropanol = 1:9, v/v); the elution gradient: 30% B for 7 min, linear gradient to 100% B over 18 min, and then 30% B held for 5 min. Mass spectrometry analysis was performed on a Q-Exactive Plus (Thermo Scientific) operating in either negative (ESI−) or positive (ESI+) electrospray ionization mode. The analysis conditions were as follow:

Positive: heater temperature 300 °C, sheath gas flow rate 45 arb, aux gas flow rate15 arb, sweep gas flow rate 1 arb, spray voltage 3.0 KV, capillary temperature 350 °C, S-lens RF level 50%. MS1 scan ranges: 200–1800.

Negative: heater temperature 300 °C, sheath gas flow rate 45 arb, aux gas flow rate 15 arb, sweep gas flow rate 1 arb, spray voltage 2.5 KV, capillary temperature 350 °C, S-lens RF level 60%. MS1 scan ranges: 250–1800.

### Data searching for lipidomics

Lipid species were identified using the LipidSearch software version 4.1 (Thermo Scientific) to process the raw data. A 5-p.p.m. mass tolerance was applied for peak alignment, retention time correction, and peak area extraction. The metabolite and lipid unidentified in >50% samples were removed.

### Bioinformatics and statistical analysis

Heatmaps were generated with the Pheatmap package in R, version 3.5.2 (R Foundation). To eliminate the influence of the order of magnitude of proteins, genes, and metabolites, we log2 transformed the intensity of them, zero-centered, and then add 1 to each value. Differential expression was assessed with a linear model and the Bioconductor limma package in R, version 3.5.2. GSEA (version 4.0.3, www.broadinstitute.org/gsea/) was used to identify GO pathways (MSigDB, version 7.1) that show an overrepresentation of upregulated or downregulated genes. Data are presented as means ± S.E.M. unless stated otherwise. As a general rule for cell-based experiments, graphs show the means ± SEM of *n* = *x* wells (*x* values are given in the figure legends) representative of a single experiment performed independently *y* times (*y* value is given in figure legends) for reproducibility. Statistical analysis was performed using GraphPad Prism 8.0 software.

## Supplementary information

Supplemental Material

## Data Availability

Proteomics (syn22761137) and lipid metabolomics (syn22761172) datasets are available at Synapse (https://www.synapse.org/#!Synapse:syn22760961). To access the data, a data use agreement is needed.
