# Supplementary material for: Thrombin induces ACSL4-dependent ferroptosis during cerebral ischemia/reperfusion
Source: Signal Transduct Target Ther. 2022 Feb 23;7:59. doi: 10.1038/s41392-022-00917-z (PMC8866433; doi:10.1038/s41392-022-00917-z)
Supplement: Supplementary file 1 — Supplemental Material [file 41392_2022_917_MOESM1_ESM.docx]

Supplementary Materials for

**Thrombin induces ACSL4-dependent ferroptosis during cerebral ischemia/reperfusion**

Qing-zhang Tuo, Yu Liu, Zheng Xiang, Hong-Fa Yan, Ting Zou, Yang Shu, Xu-long Ding, Jin-jun Zou, Shuo Xu, Fei Tang, Yan-qiu Gong, Xiao-lan Li, Yu-jie Guo, Zhao-yue Zheng, Ai-ping Deng, Zhang-zhong Yang, Wen-jing Li, Shu-ting Zhang, Scott Ayton, Ashley I. Bush, Heng Xu, Lunzhi Dai^✉^, Biao Dong^✉^, and Peng Lei^✉^

Correspondence to: Peng Lei ([peng.lei@scu.edu.cn](mailto:peng.lei@scu.edu.cn)) or Lunzhi Dai ([lunzhi.dai@scu.edu.cn](mailto:lunzhi.dai@scu.edu.cn)) or Biao Dong ([biaodong@scu.edu.cn](mailto:biaodong@scu.edu.cn))

**This PDF file includes:**

Additional Materials and Methods

Tables S1 to S3

Figures. S1 to S7

References and notes

Materials and Methods

**sgRNAs design**

sgRNAs were designed by online tools ([http://crispr.mit.edu/](http://crispr.mit.edu/guides/1999180448601928) and <https://crispr.cos.uni-heidelberg.de/>), and the off-target sites were predicted by <http://asia.ensembl.org/>. The sgRNA sequences with fewer off-target sites were selected for gene editing. The sgRNAs and their target sequences used in this study are listed in **Supplementary Table S2**. Targeting efficiency assay by T7E1 digestion is shown in **Supplementary Fig. S6**. sgRNAs for mouse ACSL4 knockout were inserted into BbsI-cleaved pssAAV-EF-gRNA to generate a series of pssAAV-mACSL4-sp.g plasmids. The sgRNA-containing plasmid and the Cas9 expression plasmid pssAAV-EF-cas9 were co-transfected into B16 cells using Lipofectamine 2000 (Life Technologies). The genomic DNAs were extracted for targeting efficiency assay (**Supplementary Fig. S6**), and the used PCR primers are listed in **Supplementary Table S3**. sgRNAs for rat ACSL4 knockout were inserted into BsaI-cleaved pX601-AAV-CMV (Addgene 61591) to generate two pX601-AAV-rACSL4-sa.g plasmids, and individual plasmid was transfected into N27 cells using Lipofectamine 2000 (Life Technologies).

**ACSL4 OE and ACSL4 KO N27 cell lines construction**

ACSL4 cDNA of the rat was introduced into SmaI-cleaved pLVX-Puro (Clontech Laboratories, 632164) to generate ACSL4 overexpressing plasmid pLenti-rACSL4. sgRNAs for rat ACSL4 knockout were inserted into BsmBI-cleaved lentiviral vector p12-2 (kindly provided by Chong Chen lab, Sichuan University). Lentiviral vectors were packaged in HEK293T by the calcium phosphate method. Briefly, the cells were cultured in a 6-well plate, and the amount of ACSL4 overexpressing or ACSL4 knockout plasmid, psPAX2 (Addgene 12260), and pMD2.G (Addgene 12259) used for transfection in each well were 4 μg, 2 μg, and 1 μg respectively. Puromycin was added to select stable cell lines at a final concentration of 1ug/ml at 48 hrs post-transfection.

**Detection of intracellular Fe^2+^**

Intracellular Fe^2+^ was detected using FerroOrange (Dojindo, Japan) as the instructions described. N27 cells were treated by OGD for 2 hrs and then incubated with FerroOrange in serum-free RPMI 1640 for 30 min in a 37 ℃ incubator equilibrated with 95% air and 5% CO_2_. The cells were observed under a fluorescence microscope.

**Intracellular ROS measurements**

Total intracellular ROS was determined by ROS Assay Kit (S0033M, Beyotime, Nantong, China), as described previously^1^. Briefly, cells were washed with PBS and incubated with 10 μM DCFH-DA at 37 °C for 30 mins. Cells were then washed twice with PBS and observed by a fluorescence microscope. All procedures were performed according to the manufacturer’s protocol.

**Real-time quantitative PCR**

Total RNA was extracted with TRIzol^TM^ reagent (15596018, Invitrogen) and reverse-transcribed to cDNA by utilizing a reverse transcription reagents kit (K1622, ThermoFisher) according to the manufacturer’s instruction. Fifty nanograms of cDNA were used to analyze gene expression by TransStart Tip Green qPCR SuperMix (AQ141-01, TransGen Biotech) on a CFX96 Real-Time PCR Detection System (Bio-Rad, USA). The primer sequences for rat ACSL4: forward primer 5’-TTTGGCTCATGTGCTGGAAC-3’, reverse primer 5’-TCACCCTTGCTTCCCTTCTT-3’; β-actin: forward primer 5’-CCTCTGAACCCTAAGGCCAA-3’, reverse primer 5’-GTCTCCGGAGTCCATCACAA-3’. The PCR cycle was as follows: 95 ℃/30 s, 40 cycles of 95 °C/5 s, 60 °C/30 s and 72 °C/30 s, and the melt-curve analysis was performed following each experiment. Fold changes were calculated with the △Ct method.

**Lipid peroxidation assay of brain tissue**

A lipid peroxidation assay kit (A106, Jiancheng, Nanjing, China) was used to test the lipid peroxidase (LPO) level in lysates of brain tissue, as described previously^2^. Briefly, the brain tissue samples were homogenized at 4 °C in 20 mM Tris buffer (pH 7.4). Then, the tissue lysates were centrifuged at 2,000 × g for 10 mins at 4 °C, and the supernatant was collected for LPO assay. Aldehydes [malondialdehyde (MDA) and hydroxynonenal (HNE)], byproducts of lipid peroxidation, react with indoles under the condition of 45 °C for 60 mins and produce diindolylalkane (a stable chromophore) with a maximum absorption peak at 586 nm.

The concentration of MDA was assessed using a lipid peroxidation MDA assay kit (S013M, Beyotime, Nantong, China), as described previously^3^. Briefly, the brain tissue samples were homogenized and sonicated in cell lysis buffer (P0013, Beyotime, Nantong, China) on ice. Then, the tissue lysates were centrifuged at 12,000 × g for 10 mins at 4 °C, and the supernatant was collected for MDA assay. Place 100 μl of the supernatant from each homogenized sample into a microcentrifuge tube. Add 200 μL of the thiobarbituric acid (TBA) solution into each tube. Incubate samples at 100 °C for 15 mins. The MDA in the sample reacted with TBA to generate an MDA–TBA adduct. Cool samples to room temperature in a water bath, then centrifuged at 1000 × g for 10 mins at room temperature. Pipette 200 mL from each reaction mixture into a 96-well plate for analysis. Measure the absorbance at 532 nm using a microplate reader.

**Transmission electron microscopy**

For rats, after ischemia 90 mins/reperfusion 24 hrs, the rat was sacrificed and the brain was washed with precooled PBS (pH = 7.4). Cut a small piece of the cortex on the ipsilateral and contralateral brain hemisphere with a sharp blade, respectively. For cells, after thrombin treatment for 24 hrs or OGD 2 hrs/reoxygenation 18 hrs, cells were collected into a 1.5 mL EP tube, in which the cells were centrifuged at low speed to the bottom of the EP tube, and the supernatants were removed. Tissues and cell pellets were immediately fixed with 0.1 M PBS (pH = 7.4) containing 2.5% glutaraldehyde for 4 hrs at 4 ℃, post-fixed in 1% osmium tetroxide for 2 hrs at room temperature (20 ℃), dehydrated in gradual ethanol (50-100%) and acetone, embedded in epoxy resin. Polymerization was performed for 48 hrs at 60 ℃. Ultrathin sections (80 nm) were cut, stained with uranyl acetate and lead citrate before transmission electron microscopy analysis (HT7700, HITACHI). Images were taken with a Slow Scan CCD camera and iTEM software (Olympus Soft Imaging Solutions).

**Western blot and analysis**

Mice and rats were deeply anesthetized using chloral hydrate (BBI Life Sciences) and transcardially perfused with PBS before the brains were removed. The cerebral cortex and hippocampus were dissected and homogenized in ice-cold lysis buffer containing 50 mM Tris-HCl (pH = 7.6), 150 mM NaCl, 1%(v/v) Triton X-100, protease inhibitor cocktail (1:50, Roche), and phosphatase inhibitors II and III (1:1000). After clearing debris by centrifuging at 14,000×g at 4℃, protein concentration in the extracts was determined by BCA Protein Assay Kit (Beyotime). Cells were collected and followed the above procedures. Aliquots of homogenate with equal protein concentrations were separated in 4–12% bis-Tris gels with NuPAGE MES running buffer (Invitrogen) and transferred to nitrocellulose membranes by iBlot2 (Invitrogen). The membranes were blocked with milk (10% v/v) and probed with appropriate primary and secondary IgG-HRP conjugated antibodies (Sigma). An enhanced chemiluminescence detection system (Thermo SCIENTIFIC) was used for development, and Bio-RAD’s ChemiDoc XRS+ system was used for visualization. Densitometry quantification of immunoreactive signals was performed by Image J (1.49m, NIH), normalized to the relative amount of β-actin, and expressed as a percentage of the mean of the control group. The following antibodies were used in this study: antibody to β-actin (1:5000, Abcam, ab179467); antibody to ACSL4 (1:1000, Abcam, ab155282), antibody to Thrombin (1:1000, Abcam, ab208590). All uncropped images of western blot are shown in **Supplementary Fig. S7**.

**Protein extraction and isobaric labeling**

The mouse brain tissues were shredded and immersed in RIPA buffer (1% NP-40, 0.5% (w/v) sodium deoxycholate, 150 mM NaCl, 50 mM Tris (pH = 7.5)) containing protease and phosphatase inhibitor, then homogenized by Gentle-MACS (Miltenyi Biotec GmbH) under the procedure “protein 01. 01” for twice, followed by 5 min sonication under the condition of 0.3 s on and 1.7 s off with 195 watts of JY92-IIN (NingBoXinYi, China). Then lysate was centrifuged at 20,000 rcf for 30 min, and the supernatant was transferred to a new tube with Bradford assay to detect protein concentration. Extracted proteins (50 μg) from each sample was reduced by Tris (2-carboxyethyl) phosphine (TCEP) with a final concentration of 10 mM at 56 ℃ for 1 hr, alkylated with iodoacetamide with a final concentration of 20 mM in the dark at room temperature for additional 30 min, and then precipitated with methanol, chloroform, and water (CH_3_OH:CHCl_3_:H_2_O = 4:1:3). After air-dried the precipitate, then digested it using sequence grade trypsin (wt/wt = 50:1) in 50mM triethylammonium bicarbonate (TEAB) buffer. The tryptic peptides of each sample were labeled with 6-plex TMT (Thermo Fisher Scientific) reagents according to the manufacturer’s protocol. After quenching with 5% hydroxylamine, TMT-labeled peptides of mouse brain samples were mixed and desalted.

For sera, the abundant proteins were removed by ProteoExtract® Albumin/IgG Removal Kit (Millipore), then the protein concentration were measured by Bradford (Bio-Rad). 50 ug proteins were lyophilized and redissolve by 6M urea, and reduced by 10 mM of TCEP at 56 °C for 1 hr, alkylated with 20 mM of IAM for 30 min to block the free cysteine residues. Then dilution to 2M urea, and digested with trypsin at 37°C in a 1:50 (w/w, trypsin/protein) ratio overnight. The peptides soaps were desalted by C18 column and labeled by 10-plex (Thermo Fisher Scientific) reagents according to the manufacturer’s protocol.

**Peptide fractionation**

The desalted TMT-labeled peptides were fractionated using reversed-phase high-performance liquid chromatography (RP-HPLC, Agilent-1260) under basic pH by reverse-phase C18 column. The mobile phase was composed of buffer A (98% H2O with 2% ACN, 10 mM ammonium formate, pH = 10) and buffer B (90% ACN with 10% H2O, 10mM ammonium formate, pH = 10). A standard 120 min LC gradient run was presented as below: 0-10 min, 0%-8% Buffer B; 10-80 min, 8%-35% Buffer B; 80-95 min, 35%-60% Buffer B; 95-105 min, 60%-70% Buffer B; 105-120 min, 70%-100% Buffer B, and the flow rate was 1 mL/min. The peptide mixture was separated into 120 fractions and combined into 40 fractions, which were dried in the speed vacuum.

**Sample preparation for lipidomics**

The samples were thawed at 4 °C and added 200 μL pre-cooled ddH_2_O. Then, the samples were homogenized, added 240 μL of cold methanol, and vortexed. Then, 800 µL MTBE was added, and the samples were mixed by the vortex. The samples were then ultra-sonicated for 20 min at a cold-water bath and then placed at room temperature for 30 min. The mixtures were centrifuged for 15 min (14000 g, 10 °C), and the upper organic phase was collected and dried with nitrogen. For LC-MS analysis, the samples were re-dissolved and vortexed in 200 μL of an isopropanol solution, vortexed, and centrifuged for 15 min (14000 g, 10 °C). The supernatants were used for further mass spectrometry analysis.

**Table S1. Demographic and clinical information for human cases.**

| **Case No.** | **Age** | **Sex** | **MoCA** | **MoCA_scale** |
| --- | --- | --- | --- | --- |
| HC1 | 56 | M | N/A |  |
| HC2 | 65 | M | N/A |  |
| HC3 | 62 | F | N/A |  |
| HC4 | 65 | F | N/A |  |
| HC5 | 64 | M | N/A |  |
| HC6 | 64 | M | N/A |  |
| HC7 | 63 | F | N/A |  |
| HC8 | 59 | F | N/A |  |
| HC9 | 62 | F | N/A |  |
| HC10 | 64 | F | N/A |  |
| HC11 | 53 | M | N/A |  |
| HC12 | 61 | M | N/A |  |
| HC13 | 65 | F | N/A |  |
| HC14 | 73 | M | N/A |  |
| HC15 | 59 | F | N/A |  |
| HC16 | 65 | F | N/A |  |
| HC17 | 58 | F | N/A |  |
| HC18 | 56 | M | N/A |  |
| HC19 | 62 | M | N/A |  |
| HC20 | 55 | M | N/A |  |
| HC21 | 66 | M | N/A |  |
| HC22 | 55 | M | N/A |  |
| HC23 | 66 | M | N/A |  |
| HC24 | 65 | F | N/A |  |
| HC25 | 55 | M | N/A |  |
| HC26 | 62 | F | N/A |  |
| HC27 | 58 | M | N/A |  |
| IS1 | 54 | M | 11 | 1 |
| IS2 | 68 | F | 24 | 0 |
| IS3 | 73 | F | 13 | 1 |
| IS4 | 60 | F | 28 | 0 |
| IS5 | 68 | F | 22 | 0 |
| IS6 | 69 | F | 24 | 0 |
| IS7 | 73 | M | 16 | 1 |
| IS8 | 57 | M | 27 | 0 |
| IS9 | 57 | M | 29 | 0 |
| IS10 | 51 | F | 27 | 0 |
| IS11 | 68 | M | 5 | 1 |
| IS12 | 60 | M | 5 | 1 |
| IS13 | 64 | M | 15 | 1 |
| IS14 | 74 | M | 22 | 0 |
| IS15 | 58 | M | 22 | 0 |
| IS16 | 64 | M | 19 | 1 |
| IS17 | 60 | F | 24 | 0 |
| IS18 | 85 | F | 16 | 1 |
| IS19 | 60 | F | 13 | 1 |
| IS20 | 79 | F | 5 | 1 |
| IS21 | 70 | F |  | 1 |
| IS22 | 88 | M |  |  |
| IS23 | 70 | F | 14 | 1 |
| IS24 | 70 | M | 10 | 1 |
| IS25 | 87 | F |  |  |
| IS26 | 52 | M |  |  |
| IS27 | 63 | M |  |  |
| IS28 | 68 | M | 12 | 1 |
| IS29 | 76 | F | 25 | 0 |
| IS30 | 74 | M | 27 | 0 |
| IS31 | 63 | F | 15 | 1 |
| IS32 | 56 | M | 14 | 1 |

Abbreviations: HC, Healthy control; IS, Ischemic stroke; M, Male; F, Female; MoCA, Montreal Cognitive Assessment; N/A, Not Applicable.

**Table S2. DNA sequences bound by sgRNAs**

| sgRNA | Target sequences | PAM |
| --- | --- | --- |
| mACSL4-sp.g1- | GGTTCTACGGGCCGCCCCAA | GGG |
| mACSL4-sp.g2 | GGCCAGTGTGAACGTATCCC | TGG |
| mACSL4-sp.g3 | GTCCAGGGATACGTTCACAC | TGG |
| rACSL4-sa.g1 | GAAGGAGTTGGTCTACTTGG | AGGAAT |
| rACSL4-sa.g2 | GGCTCTACTGGCCGCCCCAA | GGGAGT |

**Table S3. PCR primers for gene editing efficiency assay**

| **Primer** | **Sequence** |
| --- | --- |
| ACSL4-mouse-ed-F(F1） | TCAGGTGGAGCTGAAAATAGGC |
| ACSL4-mouse-ed-R(R1) | GCTGGTCCAGAACAGTATGTCA |
| ACSL4-rat-ed –F(F2) | Catccctgacctgaaccattat |
| ACSL4-rat-ed-R(R2) | AGTGTGAGTGGTGACGAATATC |


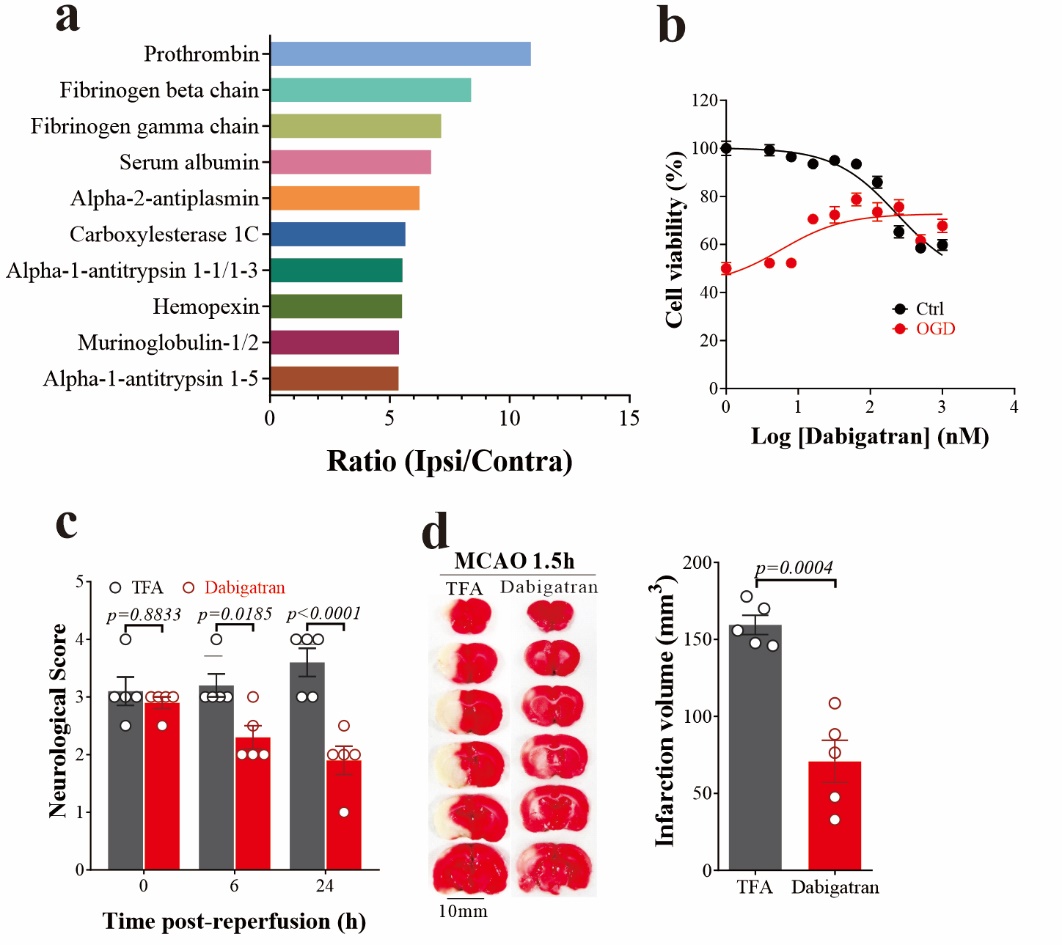


**Figure. S1. Thrombin inhibitors prevents reperfusion injury.** **a** Top ten proteins with significant changes in the ischemic hippocampus according to mass spectrometry. **b** Cell viability of N27 cells 24 hrs after OGD and Dabigatran of concentration gradient co-treatment. Data are means ± SEM, n = 6 wells from one representative of 3 independent experiments. **c** The neurological score was performed at 0, 6, and 24 hrs after MCAO/R in mice treated with Dabigatran. Data are means ± SEM, n = 5 animals per group. Two-way ANOVA with post-hoc Sidak test was performed. **d** Representative TTC-stained serial brain sections of mice 24 hrs after MCAO/R, where viable tissue stains red. Quantification of infarction volume indicated by TTC staining using Image J. Data are means ± SEM, n = 5 animals per group. t-test was performed.


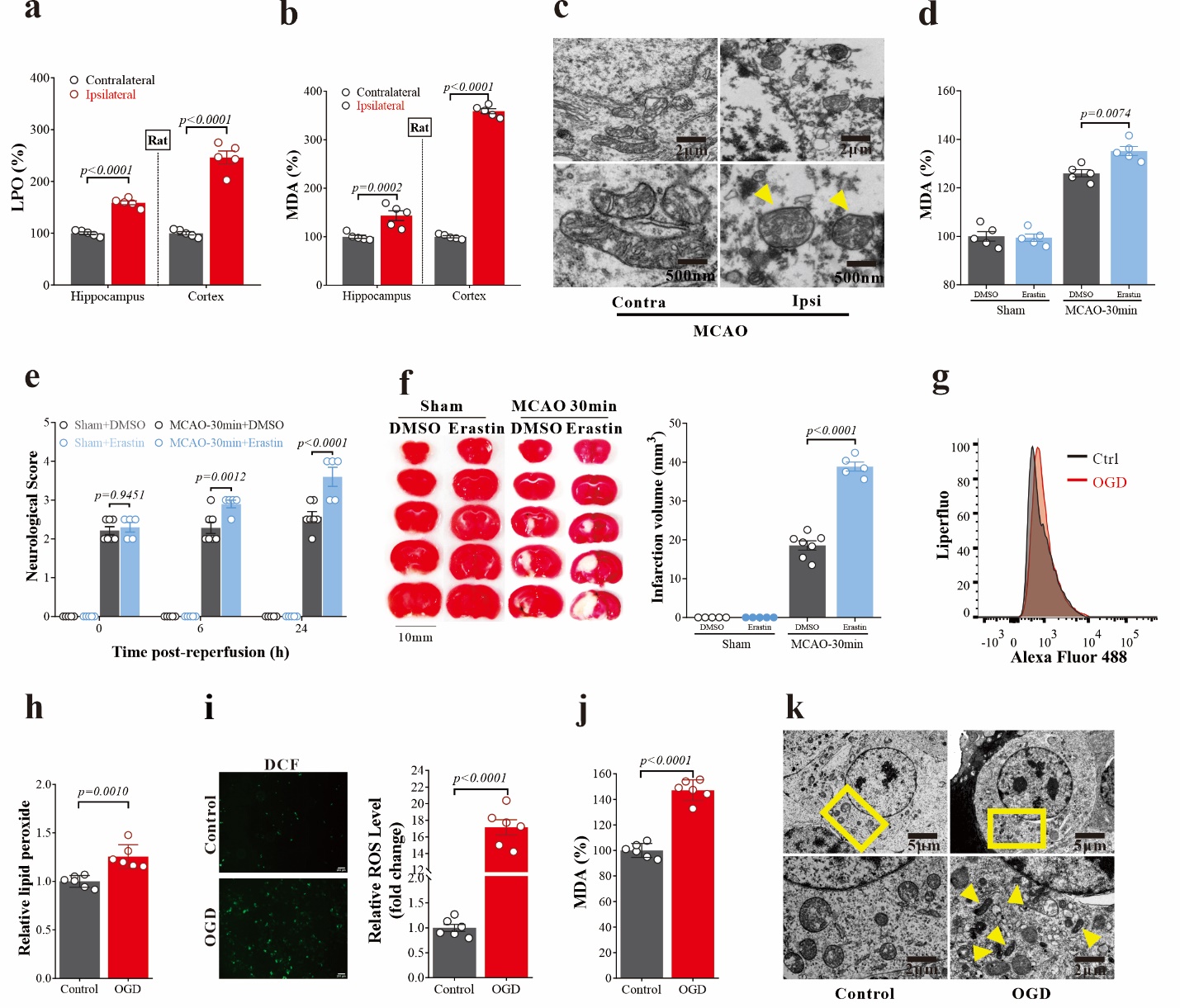


**Figure. S2. Additional data on oxygen-dependent ferroptosis in cerebral reperfusion injury.** **a** Lipid peroxidation (LPO) was detected by sensitive colorimetric assay in the hippocampus and cortex of rats following MCAO/R for 24 hrs. Data are means ± SEM, n = 5 animals per group. Two-way ANOVA with post-hoc Sidak test was performed. **b** MDA was detected by colormetric assay in the hippocampus and cortex of rats following MCAO/R for 24 hrs. Data are means ± SEM, n = 5 animals per group. Two-way ANOVA with post-hoc Sidak test was performed. **c** Representative TEM images of the rat cortex following MCAO/R for 24 hrs. Yellow arrows indicate shrunken mitochondria. **d** MDA was assayed in the hippocampus of mice following MCAO/R for 24 hrs. Data are means ± SEM, n = 5 animals per group. One-way ANOVA with post-hoc Tukey test was performed. **e** Neurological score was performed at 0, 6, and 24 hrs after MCAO/R. Data are means ± SEM. Sham, n = 5; MCAO + DMSO, n = 7; MCAO + Erastin, n = 5. Two-way ANOVA with post-hoc Tukey test was performed. **f** Representative TTC-stained serial brain sections of mice 24 hrs after MCAO/R, where viable tissue stains red. Quantification of infarction volume indicated by TTC staining using Image J. Data are means ± SEM. Sham, n = 5; MCAO + DMSO, n = 7; MCAO + Erastin, n = 5. One-way ANOVA with post-hoc Tukey test was performed. **g** Lipid peroxide in N27 cells treated with OGD for 2 hrs (representative histogram plot for fluorescence of oxidized Liperfluo). **h** Relative lipid peroxide is expressed as the ratio of oxidized to reduced Liperfluo MFI in N27 cells treated with OGD for 2 hrs. Data are means ± SEM, n = 6 wells from one representative of 3 independent experiments. t-test was performed. **i** Detection of total intracellular ROS in N27 cells by DCFH-DA staining using fluorescence microscopy (scale bar = 200 μm). Data are means ± SEM, n = 6 wells from one representative of 3 independent experiments. t-test was performed. **j** MDA was assayed in N27 cells treated with OGD for 2 hrs. Data are means ± SEM, n = 6 wells from one representative of 3 independent experiments. t-test was performed. **k** Transmission electron microscopy (TEM) of OGD-treated N27 cells. Yellow arrows indicate shrunken mitochondria.


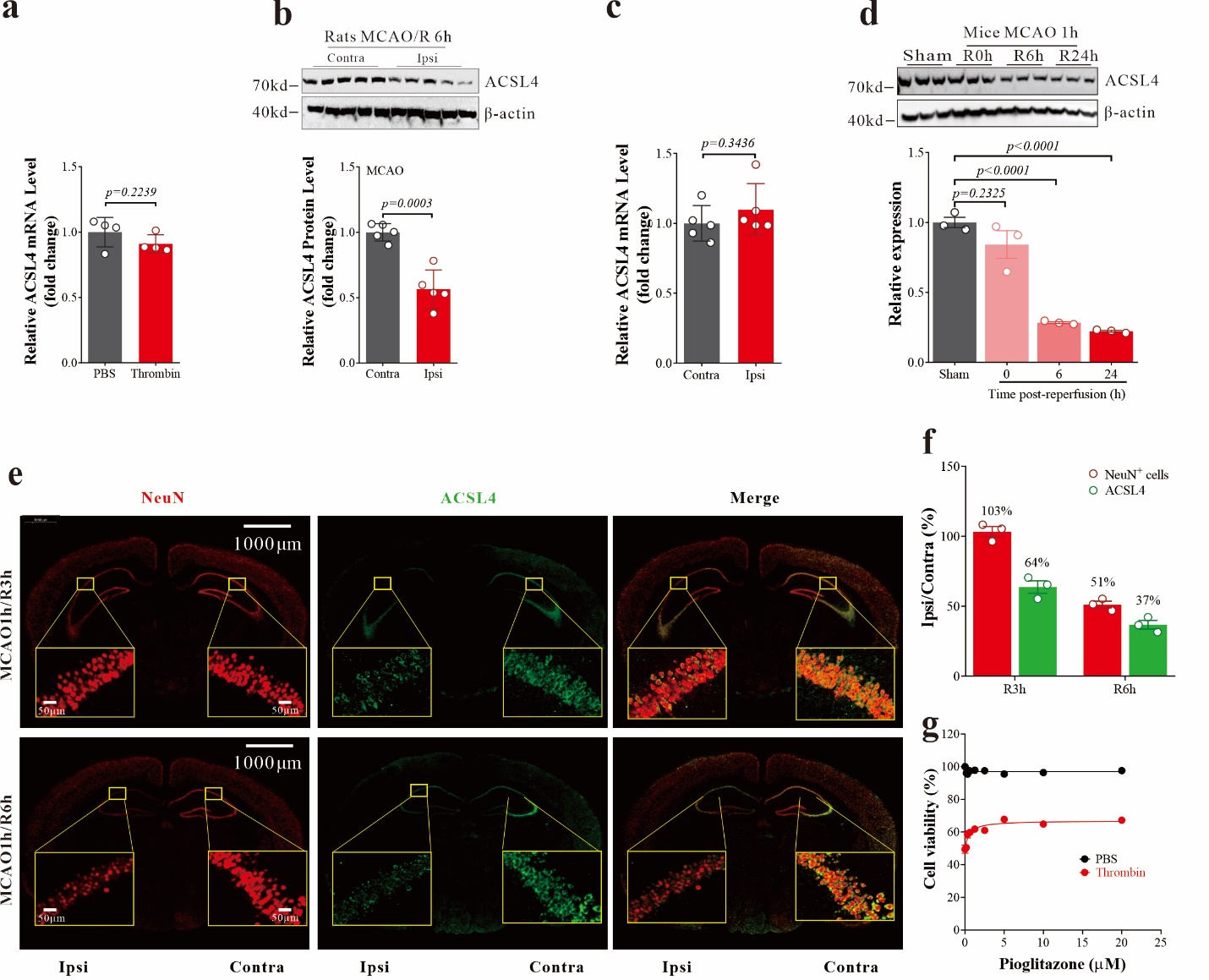


**Figure. S3. ACSL4 reduction in injured brain tissue over time post reperfusion. a** ACSL4 mRNA levels in N27 cells treated with thrombin (0.5 U/mL) for 24 hrs. Data are means ± SEM, n = 4. t-test was performed. **b** The levels of ACSL4 protein in the hippocampus on the ischemic and non-ischemic sides of the rat were examined at 6 hrs of MCAO/R. Western blots were analyzed with Image J and normalized to β-actin expression. Data are means ± SEM, n = 5 animals per group. t-test was performed. **c** qPCR analysis of ACSL4 expression in the hippocampus on the ischemic and non-ischemic sides of the rat at 6 hrs of MCAO/R. Data are means ± SEM, n = 5 animals per group. t-test was performed. **d** ACSL4 protein levels were examined from the ischemic ipsilateral hippocampus of mice that underwent MCAO after 0, 6, or 24 hrs of reperfusion. Western blots were analyzed with Image J and normalized to β-actin expression. Data are means ± SEM, n = 3 animals per group. One-way ANOVA with post-hoc Tukey test was performed. **e** Immunofluorescence staining for NeuN (red) and ACSL4 (green) from R3h (3 hrs after MCAO/R) and R6h (6 hrs after MCAO/R) mice were stained with NeuN (red) and ACSL4 (green). **f** The ratio of NeuN^+^ cell numbers and the ratio of ACSL4 immunofluorescence intensity in the ischemic ipsilateral and contralateral region of interest (ROI). (n = 3 mice per group). **g** Cell viability of N27 cells 24 hrs after thrombin (0.5 U/mL) and PIO of concentration gradient co-treatment. Data are means ± SEM, n = 5 wells from one representative of 3 independent experiments.


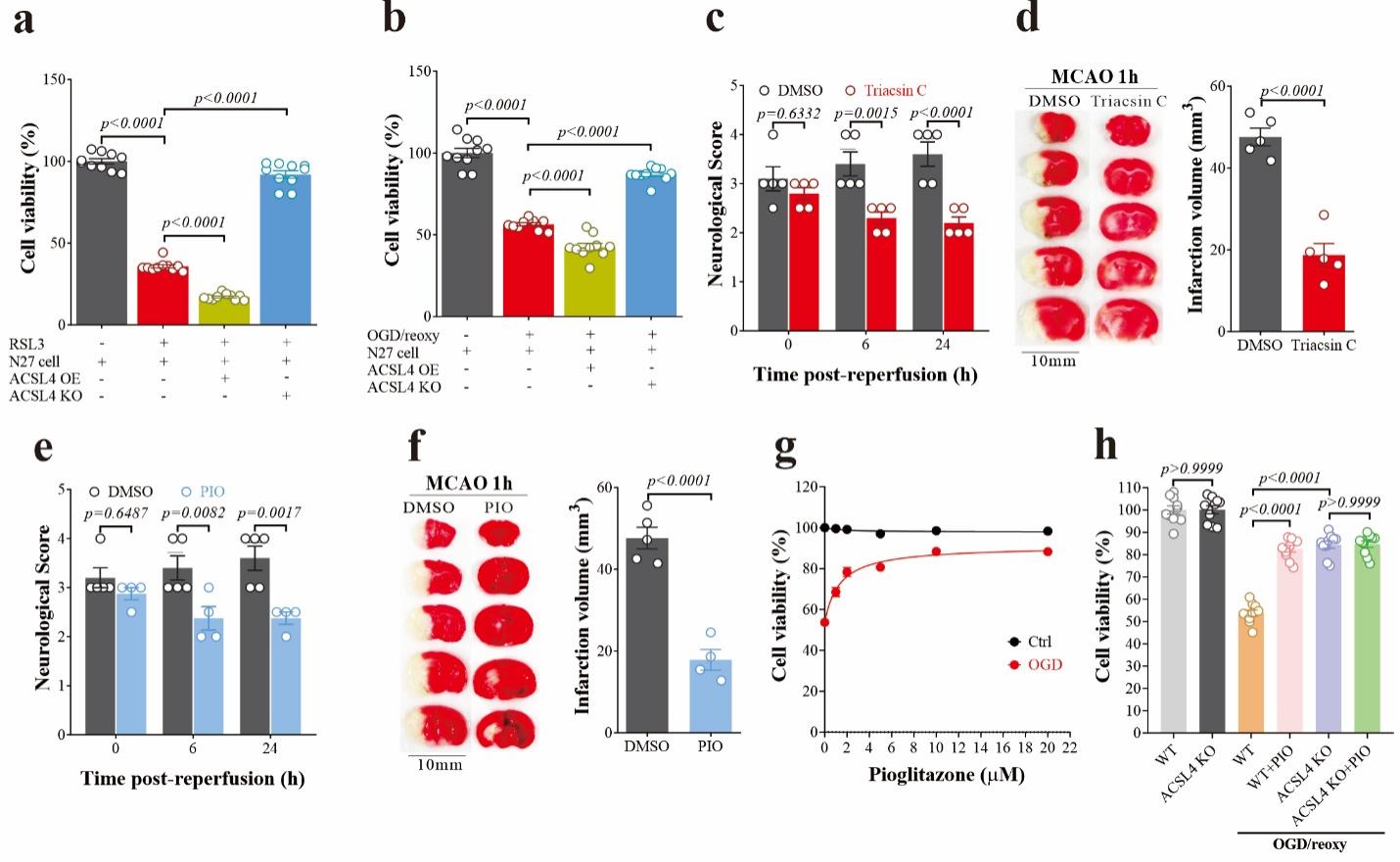


**Figure. S4. Inhibition of ACSL4 can reduce acute ischemic brain injury. a** Cell viability of WT, ACSL4 OE and ACSL4 KO N27 cells 24 hrs after RSL3 (2 μM) treatment. Data are means ± SEM, n = 10 wells from one representative of 3 independent experiments. One-way ANOVA with post-hoc Tukey test was performed. **b** Cell viability of WT, ACSL4 OE and ACSL4 KO N27 cells 18 hrs after OGD/reoxygenation. Data are means ± SEM, n = 10 wells from one representative of 3 independent experiments. One-way ANOVA with post-hoc Tukey test was performed. **c** The neurological score was performed at 0, 6, and 24 hrs after MCAO/R in mice treated with triacsin C. Data are means ± SEM, n = 5 animals per group. Two-way ANOVA with post-hoc Sidak test was performed. **d** Representative TTC-stained serial brain sections of mice 24 hrs after MCAO/reperfusion, where viable tissue stains red. Quantification of infarction volume indicated by TTC staining using Image J. Data are means ± SEM, n = 5 animals per group. t-test was performed. **e** The neurological score was performed at 0, 6, and 24 hrs after MCAO/R in mice treated with Pioglitazone (PIO). Data are means ± SEM. DMSO, n = 5; PIO, n = 4. Two-way ANOVA with post-hoc Sidak test was performed. **f** Representative TTC-stained serial brain sections of mice 24 hrs after MCAO/R, where viable tissue stains red. Quantification of infarction volume indicated by TTC staining using Image J. Data are means ± SEM. DMSO, n = 5; PIO, n = 4. t-test was performed. **g** Cell viability of N27 cells 24 hrs after OGD and PIO of concentration gradient co-treatment. Data are means ± SEM, n = 5 wells from one representative of 3 independent experiments. **h** PIO (10 μM) has no additive effect on the viability of ACSL4 KO cells treated with OGD. Data are means ± SEM, n = 10 wells from one representative of 3 independent experiments. One-way ANOVA with post-hoc Tukey test was performed.


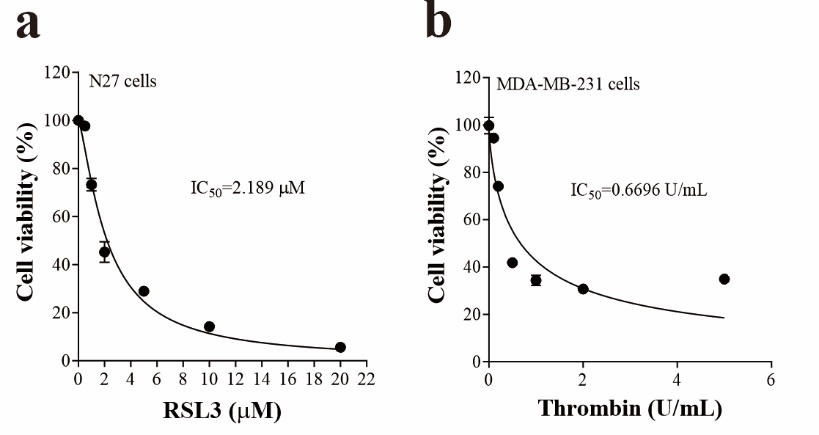


**Figure. S5. Drug cytotoxicity test. a** RSL3 cytotoxicity test in N27 cells. Data are means ± SEM, n = 5 wells from one representative of 3 independent experiments. **b** Thrombin cytotoxicity test in MDA-MB-231 cells. Data are means ± SEM, n = 6 wells from one representative of 3 independent experiments.


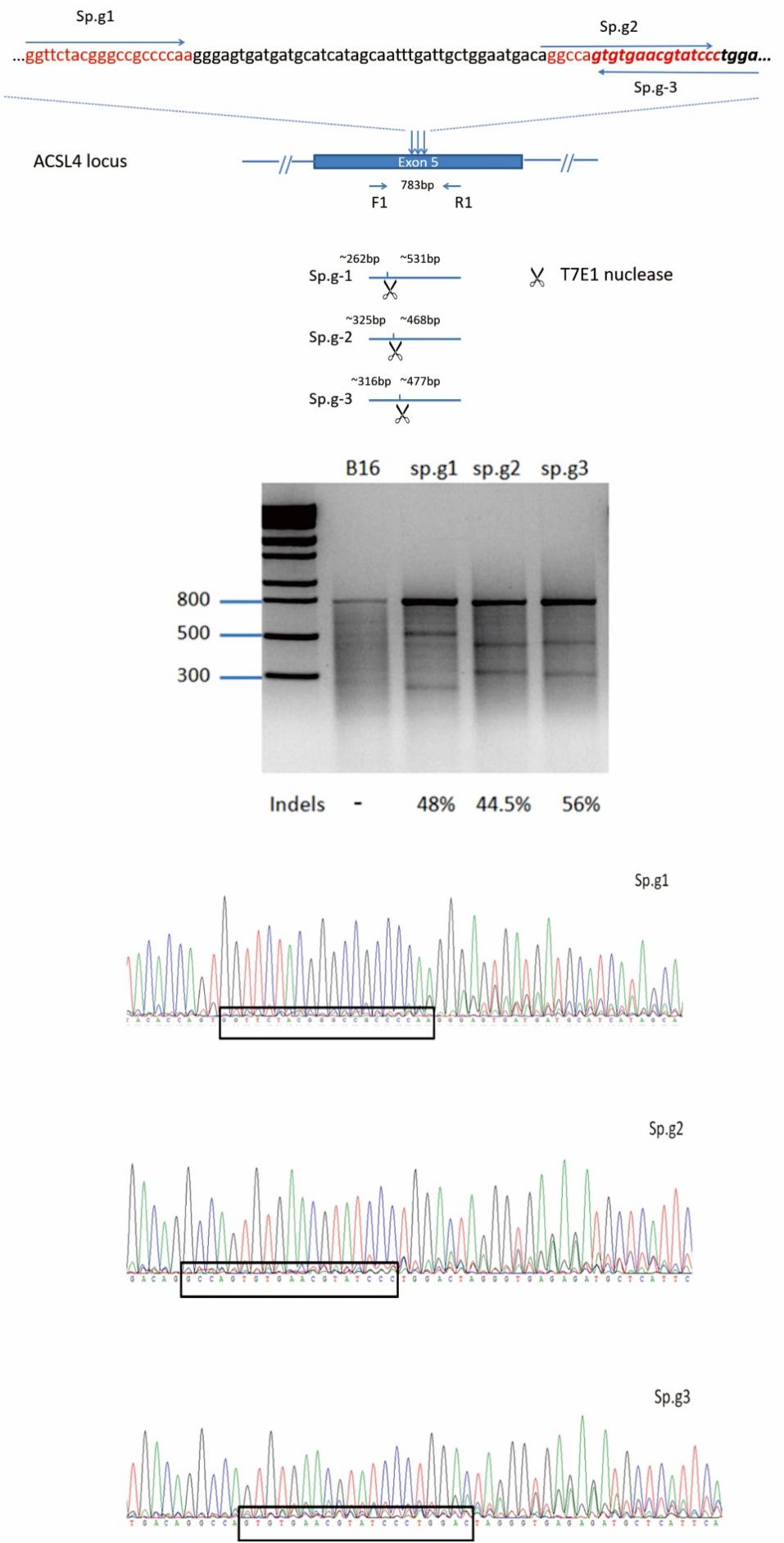


**Figure. S6. Targeting efficiency assay by T7E1 digestion.**


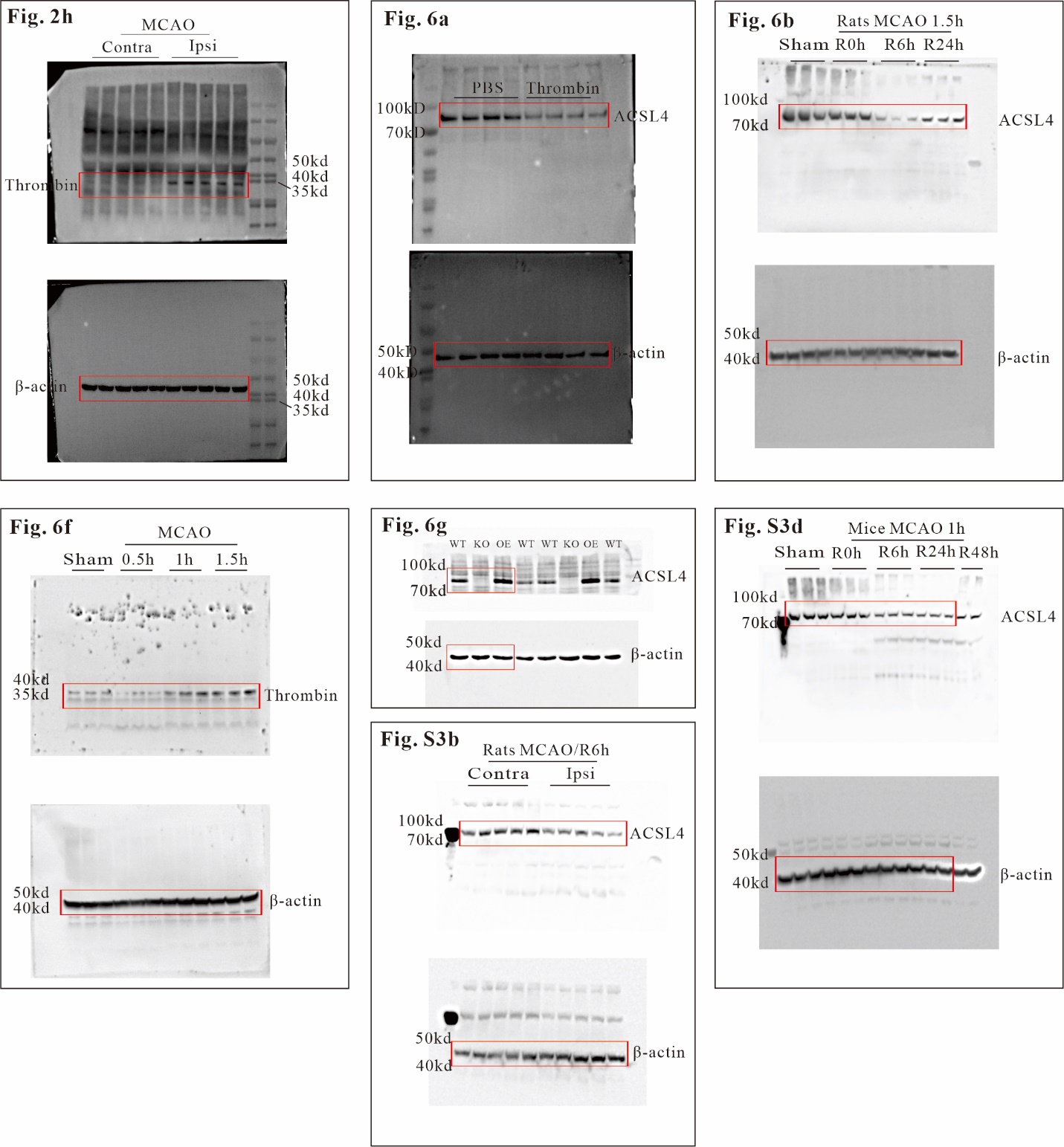


**Figure. S7. Scans of uncropped blots.**

**References and Notes**

1. Chen, X.X. *et al.* Characterization and preliminary toxicity assay of nano-titanium dioxide additive in sugar-coated chewing gum. *Small* **9**, 1765-74 (2013).

2. Li, Y. *et al.* Ischemia-induced ACSL4 activation contributes to ferroptosis-mediated tissue injury in intestinal ischemia/reperfusion. *Cell Death Differ* **26**, 2284-2299 (2019).

3. Gong, K. *et al.* Autophagy-related gene 7 (ATG7) and reactive oxygen species/extracellular signal-regulated kinase regulate tetrandrine-induced autophagy in human hepatocellular carcinoma. *J Biol Chem* **287**, 35576-88 (2012).
